# Patient, Physician, and Assessor Blinding in Phase III Randomized Trials in Oncology: A Meta‐Epidemiological Analysis

**DOI:** 10.1002/cam4.71097

**Published:** 2025-07-31

**Authors:** Gabrielle Brown, Pavlos Msaouel, Avital M. Miller, Ramez Kouzy, Timothy A. Lin, Joseph Abi Jaoude, Ethan B. Ludmir, Alexander D. Sherry

**Affiliations:** ^1^ Division of Radiation Oncology, Department of Radiation Oncology The University of Texas MD Anderson Cancer Center Houston Texas USA; ^2^ Division of Cancer Medicine, Department of Genitourinary Medical Oncology The University of Texas MD Anderson Cancer Center Houston Texas USA; ^3^ Division of Pathology and Laboratory Medicine, Department of Translational Molecular Pathology The University of Texas MD Anderson Cancer Center Houston Texas USA; ^4^ Department of Radiation Oncology and Molecular Radiation Sciences Johns Hopkins University School of Medicine Baltimore Maryland USA; ^5^ Department of Radiation Oncology Stanford University Stanford California USA; ^6^ Division of Radiation Oncology, Department of Gastrointestinal Radiation Oncology The University of Texas MD Anderson Cancer Center Houston Texas USA; ^7^ Department of Biostatistics The University of Texas MD Anderson Cancer Center Houston Texas USA; ^8^ Department of Radiation Oncology Mayo Clinic Rochester MN USA

**Keywords:** bias, blinded independent central review, double‐blind, oncology, Phase III, randomized trials

## Abstract

**Background:**

Blinding mitigates bias in randomized trials and may be especially crucial for surrogate endpoints, such as progression‐free survival (PFS). Here, we characterize utilization of and factors associated with blinding in Phase III oncology trials with PFS primary endpoints.

**Methods:**

Two‐arm, superiority‐design trials investigating systemic therapy were identified in May 2024 from ClinicalTrials.gov with no date limitation. Trials were required to have a PFS primary endpoint. The study outcomes were the presence of double‐blind designs and blinded independent central review (BICR) for the primary endpoint. Ninety‐five percent credible intervals for binary outcomes were estimated from beta distributions, and multivariable logistic regressions explored associations with trial‐level features.

**Results:**

After screening, 237 trials were included, enrolling 127,518 patients. A double‐blind design was used in 105 trials (44%, 95% CrI 38%–51%). BICR was used in 111 trials (47%, 95% CrI 41%–53%), including 39 double‐blind trials (16%, 95% CrI 12%–22%). Trials with BICR had higher odds of meeting the primary endpoint (OR 1.84; 95% CI 1.06–3.18; *p* = 0.03). The PFS assessor identity (central vs. local) or blinding status was not reported in 50 trials (26%, 95% CrI 16%–27%). Trials that met prespecified significance criteria for PFS were more likely to report whether PFS assessors were blinded/unblinded and central/local (OR, 3.05; 95% Cl: 1.60–5.81; *p* = 0.0007).

**Conclusions:**

Despite the importance of double blinding in combination with BICR for reducing bias, only a few trials blinded physicians, patients, and primary endpoint assessors. This meta‐epidemiological study illuminates the prevalence of potential assessment biases affecting PFS in Phase III oncology and secondarily emphasizes the need for improved methodology reporting.

## Graphical abstract

Blinding mitigates bias in randomized trials and may be especially crucial for surrogate endpoints, such as progression‐free survival (PFS), although it is unclear how often Phase III oncology trials utilize blinding. After reviewing 237 trials, a double‐blind design was used in 105 trials, and blinded independent central review was used in 111 trials, including in 39 double‐blind trials. Despite the importance of double blinding in combination with BICR for reducing bias, only a few trials blinded physicians, patients, and primary endpoint assessors.

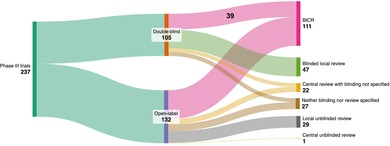

## 1 Introduction

Clinical trial design has a crucial influence on the reliable estimation of treatment effects [1]. Omission of certain design features, such as blinding, may overestimate the treatment effect [2]. Blinding may reduce patient, physician, assessor, and sponsor bias in the execution and interpretation of clinical trials. Double‐blind designs conceal the treatment allocation from both physicians and patients, which limits differential biasing behaviors due to any perceived advantages or disadvantages of the treatment allocation [3, 4]. For trials where the primary endpoint (PEP) is not overall survival but a surrogate endpoint, such as progression‐free survival (PFS), additional blinding of the disease response assessor may further reduce bias. Blinded independent central review (BICR) masks the assessor from the treatment allocation during the assessment of the treatment response. BICR may reduce biases related to the evaluation of disease by local investigators and reduce measurement variability, which may improve power to detect true treatment effects [5]. BICR is especially useful when blinding physicians and patients to the treatment allocation is not feasible. Furthermore, even when double‐blind design is used, BICR adds bias protection in scenarios when the blinding is not fully effective, such as if differing toxicity profiles can be used to surmise the identity of the treatment. Consequently, multiple regulatory agencies, including the US Food and Drug Administration (FDA), recommend BICR for registrational trials [6, 7].

Although using double‐blind designs and BICR to reduce bias is important and endorsed by regulatory agencies, the use of these tools in Phase III oncology trials has not yet been characterized. Therefore, the aim of this study was to determine the prevalence and characteristics of blinding in contemporary Phase III trials investigating PFS.

## 2 Methods

In this meta‐epidemiological study, we searched ClinicalTrials.gov to identify randomized controlled Phase III trials in oncology. The search of ClinicalTrials.gov was performed in May 2024 using the following terms: terms “cancer,” phase “Phase 3,” study results “With Results,” and status “excluded: not yet recruiting.” The quality of the underlying studies was not assessed in this meta‐epidemiological study. Inclusion criteria included two‐arm and superiority designs; furthermore, trials were required to test systemic therapy. Nonrandomized trials, cancer prevention trials, or Phase I/II studies were excluded, as well as trials in fields other than oncology. Trials without publications of the PEP, such as ongoing studies or trials presented in abstract form only, were excluded. Trials were required to have a PEP of PFS or time to progression. Trials with other PEP types, such as overall survival, were excluded. Institutional review board approval was not needed. Only publicly available data were used, and there were no individual participants or was need for informed consent.

The primary objective of this study was to assess the rate of double‐blinded designs and BICR across Phase III trials in clinical oncology investigating PFS. Double‐blinded studies were defined as those masking the intervention assignment to both the patient and the physician. Single‐blinded studies were defined as those masking the intervention to the patient alone, and open‐label studies were defined as those where both the treating physician and patient knew the identity of the treatment allocation. BICR was defined as studies assessing PFS with a blinded assessor on a centralized independent review committee, in contrast to assessment by unblinded, local radiologists or other investigators at the institution of enrollment. We differentiated between scenarios where BICR was used for the PEP analysis versus where BICR was used only as a sensitivity analysis. We also evaluated whether the methods of the PFS assessment (i.e., the blinding procedure and the identity of the assessors) were reported. Trial‐level covariates were also recorded. Rare cancers and ultra‐rare cancers were defined by a United States prevalence between 6600 and 200,000 affected citizens or less than 6600 affected citizens, respectively, based on prior literature [8]. Prevalence data were obtained for the year 2021 from the Global Burden of Disease database [9].

In order to estimate the true prevalence of these binary outcomes (double blinding and BICR) from our sample of trials, we calculated 95% credible intervals by drawing random samples from the beta distribution after 10,000 simulations, as described previously using Laplace's prior [10, 11]. The chi‐square test and Wilcoxon rank‐sum test compared trial‐level features stratified by study outcomes. In addition, the relationships between outcomes and trial variables were assessed by multivariable binary logistic regressions to estimate adjusted odds ratios (aOR). Structural causal models were created in DAGitty in order to determine confounders for each candidate predictor variable, as described in the literature (Figure S1) [12, 13]. Significance was defined as *p* < 0.05; all confidence intervals were reported at 95%, and all testing was two‐sided. Analyses were conducted using SAS v9.4 (Cary, NC) and R v4.3.2 (Vienna, Austria).

## 3 Results

After screening 418 two‐arm, superiority design, published Phase III oncology trials testing systemic therapy and excluding 181 with a PEP that was not PFS or time to progression, 237 trials were included in the analysis. These trials were published between 2006 and 2024 and enrolled a total of 127,518 patients. Of these trials, 222 (94%) were funded by industry (Table 1).

**TABLE 1:** Trial characteristics.

Characteristic	Included trials (*n* = 237)
Disease stage	
Solid nonmetastatic cancers	18 (8)
Solid metastatic cancers	163 (69)
Hematologic cancers	56 (24)
Disease site	
Breast	36 (15)
Gastrointestinal	31 (13)
Genitourinary	20 (8)
Hematologic	56 (24)
Thoracic	49 (21)
Other^a^	45 (19)
Cooperative group‐sponsored	
Yes	21 (9)
No	216 (91)
Industry‐funded	
Yes	222 (94)
No	15 (6)
Positive primary endpoint	
Yes	156 (66)
No	81 (34)
Publication year, median (IQR)	2017 (2013–2021)
Enrollment start year, median (IQR)	2011 (2007–2016)
Enrollment size, median (IQR)	466 (315–706)

*Note:* All data are *n* (%) unless otherwise specified.

Abbreviation: IQR, interquartile range.

^a^ Other cancer types included central nervous system, endocrine, gynecologic, head and neck, pediatric, sarcoma, and skin.

**FIGURE 1:**
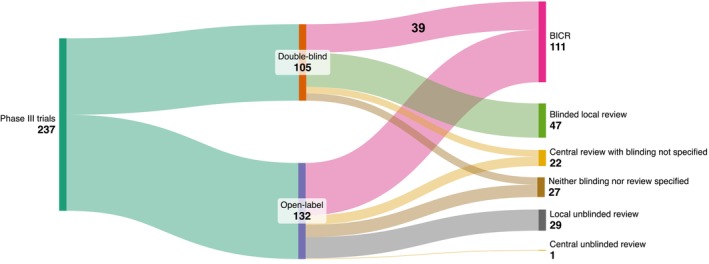
Distribution of blinding design and primary endpoint assessment methodology among Phase III oncology trials. BICR, blinded independent central review.

Of the 237 trials, 105 (44%) had used a double‐blind design, and 132 (56%) had an open‐label design (Figure 1). The estimated 95% credible interval for the true (unobserved) frequency of double‐blind studies was 38%–51%. No trials had a single‐blind design. The use of a double‐blind design did not increase over time (enrollment start year, aOR, 1.03; 95% CI: 0.97–1.08, *p* = 0.33) (Table S1). Trials studying hematologic malignancies were less likely to have double‐blind designs than trials studying metastatic solid tumors (aOR, 0.30; 95% Cl: 0.13–0.71; *p* = 0.006).

BICR was used to assess the PEP in 111 trials (47%; 95% CrI: 41%–53%), including 39 double‐blind trials (16%, 95% CrI: 12%–22%) (Figure 1). BICR use appeared to increase over time (enrollment start year, aOR, 1.10; 95% Cl: 1.04–1.61; *p* = 0.001). Cooperative groups appeared significantly less likely to use BICR designs (aOR, 0.16; 95% CI: 0.05–0.60; *p* = 0.006). Trials studying breast cancer were less likely to use BICR than trials studying all other histologic types (aOR, 0.27; 95% Cl: 0.12–0.63; *p* = 0.002) (Table S2). Trials studying rare cancers appeared to utilize BICR more often than trials investigating common cancers (58% vs. 40%) (Table S2). Trials with BICR were more likely to meet their PEP than trials without BICR (unadjusted OR, 1.84; 95% CI: 1.06–3.18; *p* = 0.03), although neither subcomponent of BICR was individually associated with a higher odds of meeting the PEP (central review unadjusted OR, 0.98; 95% CI: 0.53–1.79; *p* = 0.94; blinding unadjusted OR, 1.17; 95% CI: 0.49–2.76; *p* = 0.73). Among the remaining 76 trials where assessment methods were specified, 45 (59%) used BICR as a sensitivity analysis to complement the PEP analysis. Among these 45 trials, the overall conclusions of the BICR analysis were concordant with the overall conclusions of the primary analysis in 35 cases and discordant in one case; in the remaining nine cases, the BICR findings were not clearly reported. In total, among the 105 double‐blind trials, 72 (69%) used BICR either for the PEP analysis or as a sensitivity analysis.

Notably, 50 trials (26%; 95% CrI: 16%–27%) did not unambiguously report whether the PFS assessment was blinded and whether it was performed by local investigators or by central review (Figure 1). PFS was assessed centrally in 134 trials (57%) and locally in 76 trials (32%), but the PFS assessor identity was not specified in 27 trials (11%). Similarly, PFS assessment was blinded in 158 trials (67%) and unblinded in 30 trials (13%), but the blinding status of the PFS assessors was not specified in 49 trials (21%). Industry‐funded trials were more likely to report how PFS was assessed (blinded/unblinded vs. central/local) than nonindustry‐funded trials (184/222 [83%] vs. 4/15 [27%]; aOR, 21; 95% Cl: 5.05–90; *p* < 0.0001). In contrast, trials sponsored by a cooperative group were less likely to report the PFS assessment procedures than those not sponsored by a cooperative group (9/21 [43%] vs. 179/216 [83%]; aOR, 0.12; 95% Cl: 0.04–0.36; *p* < 0.0001). Hematologic trials were also less likely to report the PFS assessment methods (vs. metastatic solid tumor trials; aOR, 0.14; 95% Cl: 0.04–0.54; *p* = 0.004). PFS methodology transparency improved over time (enrollment start year, aOR, 1.15; 95% Cl: 1.07–1.24; *p* = 0.0002). Finally, trials that met prespecified significance criteria for PFS were more likely to report whether PFS assessors were blinded/unblinded and central/local (OR, 3.05; 95% Cl: 1.60–5.81; *p* = 0.0007).

## 4 Discussion

In this meta‐epidemiological study, we found that only a few Phase III randomized trials had double‐blind designs in combination with BICR for the primary PFS assessment. Additionally, we identified a notable lack of transparency about how PFS was measured (blinded/unblinded) and by whom (central/local). To our knowledge, this study is the first comprehensive assessment of blinding in Phase III oncology trials. Overall, this study raises awareness of the potential biases affecting measurements of PFS among Phase III trials and identifies possible opportunities for methodologic improvement.

Clinical trial design significantly influences the estimation of treatment effects. Certain design factors, such as trial size and duration, can lead to biased estimates of the treatment effects [1]. A lack of blinding may overestimate the treatment effect, particularly in trials with primary outcomes that require expert involvement to define, such as PFS [2, 14]. Although trials with an open‐label design are simpler to conduct, these trials are particularly susceptible to bias owing to both patients' and physicians' knowledge of the allocated intervention and therefore may be more difficult to interpret than those with a double‐blind design [15]. For example, patients who are disappointed with their allocated treatment may discontinue trial participation, which may lead to an informative censoring bias in the trial outcomes [16, 17, 18]. Alternatively, if physicians perceive one treatment to be superior, they may provide greater supplemental care or attention to patients allocated to that treatment or may be more likely to recommend a change in therapy to patients who are not allocated to that treatment [19]. These biases are particularly important for Phase III trials seeking to change the standard of care and receive regulatory approval.

However, implementing a double‐blind design is not always feasible, as certain treatments are more difficult to conceal from patients and/or physicians than other treatments. For example, blinding can be more challenging for patients undergoing local therapies (surgery or radiotherapy, for instance) or specific procedures differential by treatment allocation, although it is not impossible to do so, as illustrated by several sham‐controlled trials conducted with these modalities [20, 21]. Blinding may also be more challenging in rare cancer populations, although in our study, BICR was actually more prevalent among trials studying rare cancers than trials studying common cancers, suggesting that blinding is still often feasible for these populations. BICR also plays an important role in reducing bias in the evaluation of study outcomes, particularly for trials with an open‐label design, and appears to be particularly underutilized by cooperative groups [5]. However, BICR is associated with additional costs and resources and may still be hindered by other factors because radiographic progression criteria are often associated with significant interrater variability [22, 23]. Moreover, BICR can introduce bias due to informative censoring if locally evaluated progressions are unable to be confirmed centrally [5]. Thus, although BICR represents a methodological improvement compared with unblinded review, it is not a panacea for optimizing the relevance of progression‐based endpoints. Several meta‐analyses have not observed significant differences between BICR and local investigator review in the estimation of PFS treatment effects among trials that performed both types of PFS assessment; however, these analyses have been limited to a small number of trials (i.e., 27, 28, and 76 trials across three studies summing to 131 trials) [22, 24, 25, 26, 27]. Our dataset shared similar findings with concordance between the primary assessment and BICR in 34 of 35 trials (when reported). The conclusions of these studies may partially explain why, among double‐blind trials, BICR was uncommonly used for the primary PFS analysis but was more often present as a sensitivity analysis.

Despite the prevalence of BICR sensitivity analyses among double‐blind trials, however, the importance of blinding and BICR for the primary analyses becomes even more critical in pivotal registrational trials, and especially those in accelerated approval pathways, which may more immediately impact patient care. Accelerated approval is frequently on the basis of early surrogate endpoints, as shown by Downing et al. [28], when BICR is most relevant. However, blinding may even be less common among trials with indications approved in accelerated pathways, raising an additional challenge for how to interpret the effect estimations of such trials [29]. Exacerbating this concern further is the recent observation that indications approved under accelerated pathways based on surrogate endpoints often do not demonstrate improvements in overall survival or quality of life with longer follow‐up [24, 29, 30, 31]. This growing literature together implies that the current bar for accelerated approval may actually be lower than standard pathways, when it could be argued that it should be the reverse [32, 33]. Surveys of patients support this assertion, as patients often express a preference for more certain clinical benefit, such as survival or quality of life, versus faster access to novel therapies based on surrogate measures [34]. Thus, alignment of trials with patient priorities remains an evolving challenge [35]. Grassroots momentum at the trial level during design through the expanded involvement of patient advocates and methodological experts is essential for accomplishing this goal, as well as the rigorous and consistent application of standardized guidelines, such as CONSORT, at the time of trial reporting. In this vein, the Common Sense Oncology group has recently proposed a series of insightful recommendations both for trial design and reporting [36]. Physician societies such as ASCO and ESMO have also been highly valuable in advocating for these concepts and should continue to do so [37, 38]. However, to achieve more widespread effects on the trials landscape, these efforts emphasizing methodological rigor and patient‐centered metrics must be complemented by regular reexaminations of the downstream regulatory approvals process. Meta‐epidemiological studies may help facilitate this process by providing evidence on important aspects of the clinical trials landscape.

Our study had several limitations. Centralized review was based on the language each trial used and may have differed in practice somewhat between trials. There were few cooperative group studies in our dataset, limiting the generalizability in this setting [39]. The practices from the published trials in this dataset may not reflect ongoing accruing studies. In addition, trials that failed to accrue, or that have finished accrual with follow‐up, that remain unpublished may be systematically different from those that are published, and this publication bias may affect our assessment [40, 41].

In our study, despite the advantages of BICR and double‐blind trial designs, only a few trials in our study leveraged both methodological tools together, although BICR use appears to be increasing over time. Moreover, trials with BICR were more likely to achieve their primary endpoint, which may be related to multiple factors such as the overall quality of the trial, the sponsor, or the effects of BICR itself. In light of the value added by blinding and mitigation of bias, there is a greater need for trialists to consider blinding when designing their studies. Our findings emphasize the need for greater adoption of double‐blind designs, BICR, and comprehensive methods reporting among Phase III oncology trials investigating surrogate endpoints.

## Author Contributions

**Gabrielle Brown:** conceptualization (equal), data curation (equal), formal analysis (equal), investigation (equal), methodology (equal), writing – original draft (equal). **Pavlos Msaouel:** conceptualization (equal), methodology (equal), supervision (equal), writing – review and editing (equal). **Avital M. Miller:** data curation (equal), investigation (equal), writing – review and editing (equal). **Ramez Kouzy:** data curation (equal), investigation (equal), writing – review and editing (equal). **Timothy A. Lin:** data curation (equal), methodology (equal), writing – review and editing (equal). **Joseph Abi Jaoude:** data curation (equal), investigation (equal), writing – review and editing (equal). **Ethan B. Ludmir:** conceptualization (equal), data curation (equal), funding acquisition (equal), investigation (equal), methodology (equal), supervision (equal), writing – review and editing (equal). **Alexander D. Sherry:** conceptualization (equal), data curation (equal), formal analysis (equal), investigation (equal), methodology (equal), software (equal), supervision (equal), validation (equal), writing – review and editing (equal).

## Disclosure

Dr. Msaouel reports honoraria for scientific advisory board membership for Mirati Therapeutics, Bristol Myers Squibb, and Exelixis; consulting fees from Axiom Healthcare; nonbranded educational programs supported by Exelixis, Pfizer, and DAVA oncology; leadership or fiduciary roles as a Medical Steering Committee Member for the Kidney Cancer Association and as a Kidney Cancer Scientific Advisory Board Member for KCCure; and research funding from Takeda, Bristol Myers Squibb, Mirati Therapeutics, and Gateway for Cancer Research (all unrelated to this manuscript's content). Dr. Sherry reports honoraria from Sermo.

## Conflicts of Interest

The authors declare no conflicts of interest.

## Supporting information


**Appendix S1:** cam471097‐sup‐0001‐AppendixS1.docx.

## Data Availability

Research data are stored in an institutional repository and will be shared upon reasonable request to the corresponding author.
