# Supplementary material for: Patient, Physician, and Assessor Blinding in Phase III Randomized Trials in Oncology: A Meta‐Epidemiological Analysis
Source: Cancer Med. 2025 Jul 31;14(15):e71097. doi: 10.1002/cam4.71097 (PMC12313818; doi:10.1002/cam4.71097)
Supplement: Supplementary file 1 — Appendix S1: cam471097‐sup‐0001‐AppendixS1.docx. [file CAM4-14-e71097-s001.docx]

**SUPPLEMENT**

Brown et al. Patient, physician, and assessor blinding in phase III randomized trials in oncology: A meta-epidemiological analysis

**Figure S1**. Example directed acyclic graph

**Table S1**. Trial-level characteristics according to double-blind vs open-label design.

**Table S2**. Trial-level characteristics according to BICR.

**Supplemental methods.**

List of included studies.

**Figure S1**. Example directed acyclic graph illustrating the putative causal relationships between trial-level factors and the study outcomes. In this example, double-blinded design is the outcome, the predictor is in green, the confounders are in red, and the non-confounding variables are in grey. The node sponsorship was sub-divided into 2 variables: industry funding (yes versus no) and cooperative group funding (yes versus no), since trials could be funded by either, both, or neither sources.

**Table S1**. Trial-level characteristics grouped according to double-blind vs open-label design. *P* values are calculated by the chi-square test or the Wilcoxon rank-sum test.

| **Characteristic** | **Double-blind, N (%)** | **Open-label, N (%)** | ***P*** |
| --- | --- | --- | --- |
|  | n = 105 | n = 132 |  |
| **Disease stage** |  |  | 0.04 |
| Solid nonmetastatic cancers | 7 (7) | 11 (8) |  |
| Solid metastatic cancers | 81 (77) | 82 (62) |  |
| Hematologic cancers | 17 (16) | 39 (30) |  |
| **Disease site** |  |  | 0.10 |
| Breast | 17 (16) | 19 (14) |  |
| Gastrointestinal | 13 (12) | 18 (14) |  |
| Genitourinary | 7 (7) | 13 (10) |  |
| Hematologic | 17 (16) | 39 (30) |  |
| Thoracic | 26 (24) | 23 (17) |  |
| Other^a^ | 25 (25) | 20 (15) |  |
| **Prevalence** |  |  | 0.67 |
| Common | 65 (62) | 81 (62) |  |
| Rare | 40 (38) | 50 (38) |  |
| Ultra-rare | 0 (0) | 1 (0) |  |
| **Cooperative group-sponsored** |  |  | 0.13 |
| Yes | 6 (6) | 15 (11) |  |
| No | 99 (94) | 117 (89) |  |
| **Industry-funded** |  |  | 0.73 |
| Yes | 99 (94) | 123 (93) |  |
| No | 6 (6) | 9 (7) |  |
| **Positive primary endpoint** |  |  | 0.10 |
| Yes | 75 (71) | 81 (61) |  |
| No | 30 (29) | 51 (39) |  |
| **Publication year, median (IQR)** | 2017 (2013 to 2021) | 2016 (2013 to 2020) | 0.87 |
| **Enrollment start year, median (IQR)** | 2013 (2008 to 2016) | 2011 (2007 to 2016) | 0.26 |
| **Enrollment size, median (IQR)** | 521 (351 to 733) | 442 (304 to 612) | 0.049 |

Abbreviations: IQR (interquartile range). All data are n (%) unless otherwise specified.

^a^ Other cancer types included central nervous system, endocrine, gynecologic, head and neck, pediatric, sarcoma, and skin.

**Table S2**. Trial-level characteristics grouped according to the use of blinded independent central review (BICR) for the progression-free survival primary endpoint. *P* values are calculated by the chi-square test or the Wilcoxon rank-sum test.

| **Characteristic** | **BICR used, N (%)** | **BICR not used / not specified, N (%)** | ***P*** |
| --- | --- | --- | --- |
|  | n = 111 | n = 126 |  |
| **Disease stage** |  |  | 0.78 |
| Solid nonmetastatic cancers | 7 (6) | 11 (9) |  |
| Solid metastatic cancers | 77 (69) | 86 (68) |  |
| Hematologic cancers | 27 (24) | 29 (23) |  |
| **Disease site** |  |  | 0.01 |
| Breast | 8 (7) | 28 (22) |  |
| Gastrointestinal | 21 (19) | 10 (8) |  |
| Genitourinary | 9 (8) | 11 (9) |  |
| Hematologic | 27 (24) | 29 (23) |  |
| Thoracic | 22 (20) | 27 (21) |  |
| Other^a^ | 24 (22) | 21 (17) |  |
| **Prevalence** |  |  | 0.02 |
| Common | 59 (53) | 87 (70) |  |
| Rare | 52 (47) | 38 (30) |  |
| Ultra-rare | 0 | 1 (0) |  |
| **Cooperative group-sponsored** |  |  | 0.002 |
| Yes | 3 (3) | 18 (86) |  |
| No | 108 (97) | 108 (14) |  |
| **Industry-funded** |  |  | 0.0002 |
| Yes | 111 (100) | 111 (88) |  |
| No | 0 (0) | 15 (12) |  |
| **Positive primary endpoint** |  |  | 0.03 |
| Yes | 81 (73) | 75 (60) |  |
| No | 30 (27) | 51 (40) |  |
| **Publication year, median (IQR)** | 2018 (2014 to 2021) | 2015 (2012 to 2020) | 0.01 |
| **Enrollment start year, median (IQR)** | 2014 (2009 to 2017) | 2010 (2006 to 2014) | < 0.0001 |
| **Enrollment size, median (IQR)** | 442 (302 to 624) | 502 (322 to 755) | 0.06 |

Abbreviations: IQR (interquartile range). All data are n (%) unless otherwise specified.

^a^ Other cancer types included central nervous system, endocrine, gynecologic, head and neck, pediatric, sarcoma, and skin.

**Supplemental Methods**

For the multivariable models, a directed acyclic graph was created to determine confounders specific to each predictor, as shown in **Figure S1**. The following predictors were considered: disease site, disease stage, industry sponsorship, cooperative group sponsorship, enrollment start year, and enrollment size. Industry sponsorship and cooperative group sponsorship were not mutually exclusive categories, and were thus considered as separate nodes. Enrollment start year and enrollment size were analyzed as continuous variables. Based on the directed acyclic graph, the following confounders were determined for each predictor using DAGitty:

- Predictor: disease site. Confounders: None.
- Predictor: disease stage. Confounders: disease site.
- Predictor: industry funding. Confounders: disease site and stage.
- Predictor: cooperative group sponsorship. Confounders: disease site and stage.
- Predictor: enrollment size. Confounders: cooperative group, industry funding, disease stage.
- Predictor: enrollment year. Confounders: cooperative group, industry funding.

The following SAS procedures were used: proc freq (chi-square test), npar1way (Wilcoxon rank-sum test), and proc logistic (logistic regression). The code for the 95% credible interval estimation in R is publicly available at <https://github.com/adsherry2024/Analysis-code/tree/main> and begins on line 50. The specific variable names in the public code relate to a separate study: Sherry AD, Lin TA, McCaw ZR, Beck EJ, Kouzy R, Abi Jaoude J, Passy AH, Miller AM, Kupferman GS, Fuller CD, Thomas CR Jr, Koay EJ, Tang C, Msaouel P, Ludmir EB. Improving the clinical meaning of surrogate endpoints: An empirical assessment of clinical progression in phase III oncology trials. Int J Cancer. 2024 Dec 1;155(11):1939-1943.

List of included studies.^1-237^

1. Abou-Alfa GK, Macarulla T, Javle MM, et al. Ivosidenib in IDH1-mutant, chemotherapy-refractory cholangiocarcinoma (ClarIDHy): a multicentre, randomised, double-blind, placebo-controlled, phase 3 study. *Lancet Oncol*. Jun 2020;21(6):796-807. doi:10.1016/s1470-2045(20)30157-1

2. Aghajanian C, Blank SV, Goff BA, et al. OCEANS: a randomized, double-blind, placebo-controlled phase III trial of chemotherapy with or without bevacizumab in patients with platinum-sensitive recurrent epithelial ovarian, primary peritoneal, or fallopian tube cancer. *J Clin Oncol*. Jun 10 2012;30(17):2039-45. doi:10.1200/jco.2012.42.0505

3. Andtbacka RH, Kaufman HL, Collichio F, et al. Talimogene Laherparepvec Improves Durable Response Rate in Patients With Advanced Melanoma. *J Clin Oncol*. Sep 1 2015;33(25):2780-8. doi:10.1200/jco.2014.58.3377

4. Antonarakis ES, Park SH, Goh JC, et al. Pembrolizumab Plus Olaparib for Patients With Previously Treated and Biomarker-Unselected Metastatic Castration-Resistant Prostate Cancer: The Randomized, Open-Label, Phase III KEYLYNK-010 Trial. *J Clin Oncol*. Aug 1 2023;41(22):3839-3850. doi:10.1200/jco.23.00233

5. Antonia SJ, Villegas A, Daniel D, et al. Durvalumab after Chemoradiotherapy in Stage III Non-Small-Cell Lung Cancer. *N Engl J Med*. Nov 16 2017;377(20):1919-1929. doi:10.1056/NEJMoa1709937

6. Baggstrom MQ, Socinski MA, Wang XF, et al. Maintenance Sunitinib following Initial Platinum-Based Combination Chemotherapy in Advanced-Stage IIIB/IV Non-Small Cell Lung Cancer: A Randomized, Double-Blind, Placebo-Controlled Phase III Study-CALGB 30607 (Alliance). *J Thorac Oncol*. May 2017;12(5):843-849. doi:10.1016/j.jtho.2017.01.022

7. Barrios CH, Liu MC, Lee SC, et al. Phase III randomized trial of sunitinib versus capecitabine in patients with previously treated HER2-negative advanced breast cancer. *Breast Cancer Res Treat*. May 2010;121(1):121-31. doi:10.1007/s10549-010-0788-0

8. Baselga J, Campone M, Piccart M, et al. Everolimus in postmenopausal hormone-receptor-positive advanced breast cancer. *N Engl J Med*. Feb 9 2012;366(6):520-9. doi:10.1056/NEJMoa1109653

9. Baselga J, Cortés J, Kim SB, et al. Pertuzumab plus trastuzumab plus docetaxel for metastatic breast cancer. *N Engl J Med*. Jan 12 2012;366(2):109-19. doi:10.1056/NEJMoa1113216

10. Baselga J, Zamagni C, Gómez P, et al. RESILIENCE: Phase III Randomized, Double-Blind Trial Comparing Sorafenib With Capecitabine Versus Placebo With Capecitabine in Locally Advanced or Metastatic HER2-Negative Breast Cancer. *Clin Breast Cancer*. Dec 2017;17(8):585-594.e4. doi:10.1016/j.clbc.2017.05.006

11. Bashir Q, Thall PF, Milton DR, et al. Conditioning with busulfan plus melphalan versus melphalan alone before autologous haemopoietic cell transplantation for multiple myeloma: an open-label, randomised, phase 3 trial. *Lancet Haematol*. May 2019;6(5):e266-e275. doi:10.1016/s2352-3026(19)30023-7

12. Baudin E, Capdevila J, Hörsch D, et al. Treatment of advanced BP-NETS with lanreotide autogel/depot vs placebo: the phase III SPINET study. *Endocr Relat Cancer*. Sep 1 2024;31(9)doi:10.1530/erc-23-0337

13. Bauer S, Jones RL, Blay JY, et al. Ripretinib Versus Sunitinib in Patients With Advanced Gastrointestinal Stromal Tumor After Treatment With Imatinib (INTRIGUE): A Randomized, Open-Label, Phase III Trial. *J Clin Oncol*. Dec 1 2022;40(34):3918-3928. doi:10.1200/jco.22.00294

14. Bepler G, Williams C, Schell MJ, et al. Randomized international phase III trial of ERCC1 and RRM1 expression-based chemotherapy versus gemcitabine/carboplatin in advanced non-small-cell lung cancer. *J Clin Oncol*. Jul 1 2013;31(19):2404-12. doi:10.1200/jco.2012.46.9783

15. Bergh J, Jönsson PE, Lidbrink EK, et al. FACT: an open-label randomized phase III study of fulvestrant and anastrozole in combination compared with anastrozole alone as first-line therapy for patients with receptor-positive postmenopausal breast cancer. *J Clin Oncol*. Jun 1 2012;30(16):1919-25. doi:10.1200/jco.2011.38.1095

16. Bidard FC, Kaklamani VG, Neven P, et al. Elacestrant (oral selective estrogen receptor degrader) Versus Standard Endocrine Therapy for Estrogen Receptor-Positive, Human Epidermal Growth Factor Receptor 2-Negative Advanced Breast Cancer: Results From the Randomized Phase III EMERALD Trial. *J Clin Oncol*. Oct 1 2022;40(28):3246-3256. doi:10.1200/jco.22.00338

17. Blackwell KL, Burstein HJ, Storniolo AM, et al. Randomized study of Lapatinib alone or in combination with trastuzumab in women with ErbB2-positive, trastuzumab-refractory metastatic breast cancer. *J Clin Oncol*. Mar 1 2010;28(7):1124-30. doi:10.1200/jco.2008.21.4437

18. Blay JY, Serrano C, Heinrich MC, et al. Ripretinib in patients with advanced gastrointestinal stromal tumours (INVICTUS): a double-blind, randomised, placebo-controlled, phase 3 trial. *Lancet Oncol*. Jul 2020;21(7):923-934. doi:10.1016/s1470-2045(20)30168-6

19. Blay JY, Shen L, Kang YK, et al. Nilotinib versus imatinib as first-line therapy for patients with unresectable or metastatic gastrointestinal stromal tumours (ENESTg1): a randomised phase 3 trial. *Lancet Oncol*. May 2015;16(5):550-60. doi:10.1016/s1470-2045(15)70105-1

20. Brose MS, Capdevila J, Elisei R, et al. Vandetanib in locally advanced or metastatic differentiated thyroid cancer refractory to radioiodine therapy. *Endocr Relat Cancer*. Aug 1 2024;31(8)doi:10.1530/erc-23-0354

21. Brose MS, Robinson B, Sherman SI, et al. Cabozantinib for radioiodine-refractory differentiated thyroid cancer (COSMIC-311): a randomised, double-blind, placebo-controlled, phase 3 trial. *Lancet Oncol*. Aug 2021;22(8):1126-1138. doi:10.1016/s1470-2045(21)00332-6

22. Brufsky AM, Hurvitz S, Perez E, et al. RIBBON-2: a randomized, double-blind, placebo-controlled, phase III trial evaluating the efficacy and safety of bevacizumab in combination with chemotherapy for second-line treatment of human epidermal growth factor receptor 2-negative metastatic breast cancer. *J Clin Oncol*. Nov 10 2011;29(32):4286-93. doi:10.1200/jco.2010.34.1255

23. Burger JA, Tedeschi A, Barr PM, et al. Ibrutinib as Initial Therapy for Patients with Chronic Lymphocytic Leukemia. *N Engl J Med*. Dec 17 2015;373(25):2425-37. doi:10.1056/NEJMoa1509388

24. Burtness B, Harrington KJ, Greil R, et al. Pembrolizumab alone or with chemotherapy versus cetuximab with chemotherapy for recurrent or metastatic squamous cell carcinoma of the head and neck (KEYNOTE-048): a randomised, open-label, phase 3 study. *Lancet*. Nov 23 2019;394(10212):1915-1928. doi:10.1016/s0140-6736(19)32591-7

25. Byrd JC, Brown JR, O'Brien S, et al. Ibrutinib versus ofatumumab in previously treated chronic lymphoid leukemia. *N Engl J Med*. Jul 17 2014;371(3):213-23. doi:10.1056/NEJMoa1400376

26. Camidge DR, Kim HR, Ahn MJ, et al. Brigatinib versus Crizotinib in ALK-Positive Non-Small-Cell Lung Cancer. *N Engl J Med*. Nov 22 2018;379(21):2027-2039. doi:10.1056/NEJMoa1810171

27. Caplin ME, Pavel M, Ćwikła JB, et al. Lanreotide in metastatic enteropancreatic neuroendocrine tumors. *N Engl J Med*. Jul 17 2014;371(3):224-33. doi:10.1056/NEJMoa1316158

28. Cappuzzo F, Ciuleanu T, Stelmakh L, et al. Erlotinib as maintenance treatment in advanced non-small-cell lung cancer: a multicentre, randomised, placebo-controlled phase 3 study. *Lancet Oncol*. Jun 2010;11(6):521-9. doi:10.1016/s1470-2045(10)70112-1

29. Carbone DP, Reck M, Paz-Ares L, et al. First-Line Nivolumab in Stage IV or Recurrent Non-Small-Cell Lung Cancer. *N Engl J Med*. Jun 22 2017;376(25):2415-2426. doi:10.1056/NEJMoa1613493

30. Carrato A, Swieboda-Sadlej A, Staszewska-Skurczynska M, et al. Fluorouracil, leucovorin, and irinotecan plus either sunitinib or placebo in metastatic colorectal cancer: a randomized, phase III trial. *J Clin Oncol*. Apr 1 2013;31(10):1341-7. doi:10.1200/jco.2012.45.1930

31. Carvajal RD, Piperno-Neumann S, Kapiteijn E, et al. Selumetinib in Combination With Dacarbazine in Patients With Metastatic Uveal Melanoma: A Phase III, Multicenter, Randomized Trial (SUMIT). *J Clin Oncol*. Apr 20 2018;36(12):1232-1239. doi:10.1200/jco.2017.74.1090

32. Chanan-Khan A, Cramer P, Demirkan F, et al. Ibrutinib combined with bendamustine and rituximab compared with placebo, bendamustine, and rituximab for previously treated chronic lymphocytic leukaemia or small lymphocytic lymphoma (HELIOS): a randomised, double-blind, phase 3 study. *Lancet Oncol*. Feb 2016;17(2):200-211. doi:10.1016/s1470-2045(15)00465-9

33. Cheng Y, Zhang L, Hu J, et al. Pembrolizumab Plus Chemotherapy for Chinese Patients With Metastatic Squamous NSCLC in KEYNOTE-407. *JTO Clin Res Rep*. Oct 2021;2(10):100225. doi:10.1016/j.jtocrr.2021.100225

34. Chi KN, Agarwal N, Bjartell A, et al. Apalutamide for Metastatic, Castration-Sensitive Prostate Cancer. *N Engl J Med*. Jul 4 2019;381(1):13-24. doi:10.1056/NEJMoa1903307

35. Chinot OL, Wick W, Mason W, et al. Bevacizumab plus radiotherapy-temozolomide for newly diagnosed glioblastoma. *N Engl J Med*. Feb 20 2014;370(8):709-22. doi:10.1056/NEJMoa1308345

36. Cho BC, Ahn MJ, Kang JH, et al. Lazertinib Versus Gefitinib as First-Line Treatment in Patients With EGFR-Mutated Advanced Non-Small-Cell Lung Cancer: Results From LASER301. *J Clin Oncol*. Sep 10 2023;41(26):4208-4217. doi:10.1200/jco.23.00515

37. Cho BC, Lee JS, Wu YL, et al. Bintrafusp Alfa Versus Pembrolizumab in Patients With Treatment-Naive, Programmed Death-Ligand 1-High Advanced NSCLC: A Randomized, Open-Label, Phase 3 Trial. *J Thorac Oncol*. Dec 2023;18(12):1731-1742. doi:10.1016/j.jtho.2023.08.018

38. Choueiri TK, Escudier B, Powles T, et al. Cabozantinib versus Everolimus in Advanced Renal-Cell Carcinoma. *N Engl J Med*. Nov 5 2015;373(19):1814-23. doi:10.1056/NEJMoa1510016

39. Choueiri TK, Heng DYC, Lee JL, et al. Efficacy of Savolitinib vs Sunitinib in Patients With MET-Driven Papillary Renal Cell Carcinoma: The SAVOIR Phase 3 Randomized Clinical Trial. *JAMA Oncol*. Aug 1 2020;6(8):1247-1255. doi:10.1001/jamaoncol.2020.2218

40. Chung HC, Kang YK, Chen Z, et al. Pembrolizumab versus paclitaxel for previously treated advanced gastric or gastroesophageal junction cancer (KEYNOTE-063): A randomized, open-label, phase 3 trial in Asian patients. *Cancer*. Mar 1 2022;128(5):995-1003. doi:10.1002/cncr.34019

41. Ciuleanu T, Brodowicz T, Zielinski C, et al. Maintenance pemetrexed plus best supportive care versus placebo plus best supportive care for non-small-cell lung cancer: a randomised, double-blind, phase 3 study. *Lancet*. Oct 24 2009;374(9699):1432-40. doi:10.1016/s0140-6736(09)61497-5

42. Clarke NW, Armstrong AJ, Thiery-Vuillemin A, et al. Abiraterone and Olaparib for Metastatic Castration-Resistant Prostate Cancer. *NEJM Evid*. Sep 2022;1(9):EVIDoa2200043. doi:10.1056/EVIDoa2200043

43. Connolly RM, Zhao F, Miller KD, et al. E2112: Randomized Phase III Trial of Endocrine Therapy Plus Entinostat or Placebo in Hormone Receptor-Positive Advanced Breast Cancer. A Trial of the ECOG-ACRIN Cancer Research Group. *J Clin Oncol*. Oct 1 2021;39(28):3171-3181. doi:10.1200/jco.21.00944

44. Cortés J, Hurvitz SA, O'Shaughnessy J, et al. Randomized Phase III Study of Amcenestrant Plus Palbociclib Versus Letrozole Plus Palbociclib in Estrogen Receptor-Positive, Human Epidermal Growth Factor Receptor 2-Negative Advanced Breast Cancer: Primary Results From AMEERA-5. *J Clin Oncol*. Aug 1 2024;42(22):2680-2690. doi:10.1200/jco.23.02036

45. Cortés J, Kim SB, Chung WP, et al. Trastuzumab Deruxtecan versus Trastuzumab Emtansine for Breast Cancer. *N Engl J Med*. Mar 24 2022;386(12):1143-1154. doi:10.1056/NEJMoa2115022

46. Crown JP, Diéras V, Staroslawska E, et al. Phase III trial of sunitinib in combination with capecitabine versus capecitabine monotherapy for the treatment of patients with pretreated metastatic breast cancer. *J Clin Oncol*. Aug 10 2013;31(23):2870-8. doi:10.1200/jco.2012.43.3391

47. Cunningham D, Lang I, Marcuello E, et al. Bevacizumab plus capecitabine versus capecitabine alone in elderly patients with previously untreated metastatic colorectal cancer (AVEX): an open-label, randomised phase 3 trial. *Lancet Oncol*. Oct 2013;14(11):1077-1085. doi:10.1016/s1470-2045(13)70154-2

48. de Boer RH, Arrieta Ó, Yang CH, et al. Vandetanib plus pemetrexed for the second-line treatment of advanced non-small-cell lung cancer: a randomized, double-blind phase III trial. *J Clin Oncol*. Mar 10 2011;29(8):1067-74. doi:10.1200/jco.2010.29.5717

49. de Langen AJ, Johnson ML, Mazieres J, et al. Sotorasib versus docetaxel for previously treated non-small-cell lung cancer with KRAS(G12C) mutation: a randomised, open-label, phase 3 trial. *Lancet*. Mar 4 2023;401(10378):733-746. doi:10.1016/s0140-6736(23)00221-0

50. Demetri GD, Reichardt P, Kang YK, et al. Efficacy and safety of regorafenib for advanced gastrointestinal stromal tumours after failure of imatinib and sunitinib (GRID): an international, multicentre, randomised, placebo-controlled, phase 3 trial. *Lancet*. Jan 26 2013;381(9863):295-302. doi:10.1016/s0140-6736(12)61857-1

51. Demetri GD, van Oosterom AT, Garrett CR, et al. Efficacy and safety of sunitinib in patients with advanced gastrointestinal stromal tumour after failure of imatinib: a randomised controlled trial. *Lancet*. Oct 14 2006;368(9544):1329-38. doi:10.1016/s0140-6736(06)69446-4

52. Dent S, Cortés J, Im YH, et al. Phase III randomized study of taselisib or placebo with fulvestrant in estrogen receptor-positive, PIK3CA-mutant, HER2-negative, advanced breast cancer: the SANDPIPER trial. *Ann Oncol*. Feb 2021;32(2):197-207. doi:10.1016/j.annonc.2020.10.596

53. Di Leo A, Gomez HL, Aziz Z, et al. Phase III, double-blind, randomized study comparing lapatinib plus paclitaxel with placebo plus paclitaxel as first-line treatment for metastatic breast cancer. *J Clin Oncol*. Dec 1 2008;26(34):5544-52. doi:10.1200/jco.2008.16.2578

54. Di Leo A, Jerusalem G, Petruzelka L, et al. Results of the CONFIRM phase III trial comparing fulvestrant 250 mg with fulvestrant 500 mg in postmenopausal women with estrogen receptor-positive advanced breast cancer. *J Clin Oncol*. Oct 20 2010;28(30):4594-600. doi:10.1200/jco.2010.28.8415

55. Diab A, Gogas H, Sandhu S, et al. Bempegaldesleukin Plus Nivolumab in Untreated Advanced Melanoma: The Open-Label, Phase III PIVOT IO 001 Trial Results. *J Clin Oncol*. Oct 20 2023;41(30):4756-4767. doi:10.1200/jco.23.00172

56. Dickler MN, Barry WT, Cirrincione CT, et al. Phase III Trial Evaluating Letrozole As First-Line Endocrine Therapy With or Without Bevacizumab for the Treatment of Postmenopausal Women With Hormone Receptor-Positive Advanced-Stage Breast Cancer: CALGB 40503 (Alliance). *J Clin Oncol*. Aug 1 2016;34(22):2602-9. doi:10.1200/jco.2015.66.1595

57. Diéras V, Han HS, Kaufman B, et al. Veliparib with carboplatin and paclitaxel in BRCA-mutated advanced breast cancer (BROCADE3): a randomised, double-blind, placebo-controlled, phase 3 trial. *Lancet Oncol*. Oct 2020;21(10):1269-1282. doi:10.1016/s1470-2045(20)30447-2

58. Dimopoulos M, Quach H, Mateos MV, et al. Carfilzomib, dexamethasone, and daratumumab versus carfilzomib and dexamethasone for patients with relapsed or refractory multiple myeloma (CANDOR): results from a randomised, multicentre, open-label, phase 3 study. *Lancet*. Jul 18 2020;396(10245):186-197. doi:10.1016/s0140-6736(20)30734-0

59. Dimopoulos M, Siegel DS, Lonial S, et al. Vorinostat or placebo in combination with bortezomib in patients with multiple myeloma (VANTAGE 088): a multicentre, randomised, double-blind study. *Lancet Oncol*. Oct 2013;14(11):1129-1140. doi:10.1016/s1470-2045(13)70398-x

60. Dimopoulos M, Spencer A, Attal M, et al. Lenalidomide plus dexamethasone for relapsed or refractory multiple myeloma. *N Engl J Med*. Nov 22 2007;357(21):2123-32. doi:10.1056/NEJMoa070594

61. Dimopoulos MA, Moreau P, Palumbo A, et al. Carfilzomib and dexamethasone versus bortezomib and dexamethasone for patients with relapsed or refractory multiple myeloma (ENDEAVOR): a randomised, phase 3, open-label, multicentre study. *Lancet Oncol*. Jan 2016;17(1):27-38. doi:10.1016/s1470-2045(15)00464-7

62. Dimopoulos MA, Richardson PG, Bahlis NJ, et al. Addition of elotuzumab to lenalidomide and dexamethasone for patients with newly diagnosed, transplantation ineligible multiple myeloma (ELOQUENT-1): an open-label, multicentre, randomised, phase 3 trial. *Lancet Haematol*. Jun 2022;9(6):e403-e414. doi:10.1016/s2352-3026(22)00103-x

63. Dimopoulos MA, Špička I, Quach H, et al. Ixazomib as Postinduction Maintenance for Patients With Newly Diagnosed Multiple Myeloma Not Undergoing Autologous Stem Cell Transplantation: The Phase III TOURMALINE-MM4 Trial. *J Clin Oncol*. Dec 1 2020;38(34):4030-4041. doi:10.1200/jco.20.02060

64. Dimopoulos MA, Tedeschi A, Trotman J, et al. Phase 3 Trial of Ibrutinib plus Rituximab in Waldenström's Macroglobulinemia. *N Engl J Med*. Jun 21 2018;378(25):2399-2410. doi:10.1056/NEJMoa1802917

65. Dimopoulos MA, Terpos E, Boccadoro M, et al. Daratumumab plus pomalidomide and dexamethasone versus pomalidomide and dexamethasone alone in previously treated multiple myeloma (APOLLO): an open-label, randomised, phase 3 trial. *Lancet Oncol*. Jun 2021;22(6):801-812. doi:10.1016/s1470-2045(21)00128-5

66. DiSilvestro PA, Ali S, Craighead PS, et al. Phase III randomized trial of weekly cisplatin and irradiation versus cisplatin and tirapazamine and irradiation in stages IB2, IIA, IIB, IIIB, and IVA cervical carcinoma limited to the pelvis: a Gynecologic Oncology Group study. *J Clin Oncol*. Feb 10 2014;32(5):458-64. doi:10.1200/jco.2013.51.4265

67. Douillard JY, Siena S, Cassidy J, et al. Randomized, phase III trial of panitumumab with infusional fluorouracil, leucovorin, and oxaliplatin (FOLFOX4) versus FOLFOX4 alone as first-line treatment in patients with previously untreated metastatic colorectal cancer: the PRIME study. *J Clin Oncol*. Nov 1 2010;28(31):4697-705. doi:10.1200/jco.2009.27.4860

68. Dresemann G, Weller M, Rosenthal MA, et al. Imatinib in combination with hydroxyurea versus hydroxyurea alone as oral therapy in patients with progressive pretreated glioblastoma resistant to standard dose temozolomide. *J Neurooncol*. Feb 2010;96(3):393-402. doi:10.1007/s11060-009-9976-3

69. Dreyling M, Jurczak W, Jerkeman M, et al. Ibrutinib versus temsirolimus in patients with relapsed or refractory mantle-cell lymphoma: an international, randomised, open-label, phase 3 study. *Lancet*. Feb 20 2016;387(10020):770-8. doi:10.1016/s0140-6736(15)00667-4

70. du Bois A, Floquet A, Kim JW, et al. Incorporation of pazopanib in maintenance therapy of ovarian cancer. *J Clin Oncol*. Oct 20 2014;32(30):3374-82. doi:10.1200/jco.2014.55.7348

71. Dueñas-González A, Zarbá JJ, Patel F, et al. Phase III, open-label, randomized study comparing concurrent gemcitabine plus cisplatin and radiation followed by adjuvant gemcitabine and cisplatin versus concurrent cisplatin and radiation in patients with stage IIB to IVA carcinoma of the cervix. *J Clin Oncol*. May 1 2011;29(13):1678-85. doi:10.1200/jco.2009.25.9663

72. Dummer R, Schadendorf D, Ascierto PA, et al. Binimetinib versus dacarbazine in patients with advanced NRAS-mutant melanoma (NEMO): a multicentre, open-label, randomised, phase 3 trial. *Lancet Oncol*. Apr 2017;18(4):435-445. doi:10.1016/s1470-2045(17)30180-8

73. Durie BGM, Hoering A, Abidi MH, et al. Bortezomib with lenalidomide and dexamethasone versus lenalidomide and dexamethasone alone in patients with newly diagnosed myeloma without intent for immediate autologous stem-cell transplant (SWOG S0777): a randomised, open-label, phase 3 trial. *Lancet*. Feb 4 2017;389(10068):519-527. doi:10.1016/s0140-6736(16)31594-x

74. Eastham JA, Heller G, Halabi S, et al. Cancer and Leukemia Group B 90203 (Alliance): Radical Prostatectomy With or Without Neoadjuvant Chemohormonal Therapy in Localized, High-Risk Prostate Cancer. *J Clin Oncol*. Sep 10 2020;38(26):3042-3050. doi:10.1200/jco.20.00315

75. Elisei R, Schlumberger MJ, Müller SP, et al. Cabozantinib in progressive medullary thyroid cancer. *J Clin Oncol*. Oct 10 2013;31(29):3639-46. doi:10.1200/jco.2012.48.4659

76. Elter T, Gercheva-Kyuchukova L, Pylylpenko H, et al. Fludarabine plus alemtuzumab versus fludarabine alone in patients with previously treated chronic lymphocytic leukaemia: a randomised phase 3 trial. *Lancet Oncol*. Dec 2011;12(13):1204-13. doi:10.1016/s1470-2045(11)70242-x

77. Facon T, Venner CP, Bahlis NJ, et al. Oral ixazomib, lenalidomide, and dexamethasone for transplant-ineligible patients with newly diagnosed multiple myeloma. *Blood*. Jul 1 2021;137(26):3616-3628. doi:10.1182/blood.2020008787

78. Finn RS, Qin S, Ikeda M, et al. Atezolizumab plus Bevacizumab in Unresectable Hepatocellular Carcinoma. *N Engl J Med*. May 14 2020;382(20):1894-1905. doi:10.1056/NEJMoa1915745

79. Fizazi K, Pagliaro L, Laplanche A, et al. Personalised chemotherapy based on tumour marker decline in poor prognosis germ-cell tumours (GETUG 13): a phase 3, multicentre, randomised trial. *Lancet Oncol*. Dec 2014;15(13):1442-1450. doi:10.1016/s1470-2045(14)70490-5

80. Fizazi K, Tran N, Fein L, et al. Abiraterone plus Prednisone in Metastatic, Castration-Sensitive Prostate Cancer. *N Engl J Med*. Jul 27 2017;377(4):352-360. doi:10.1056/NEJMoa1704174

81. Flaherty KT, Robert C, Hersey P, et al. Improved survival with MEK inhibition in BRAF-mutated melanoma. *N Engl J Med*. Jul 12 2012;367(2):107-14. doi:10.1056/NEJMoa1203421

82. Furman RR, Sharman JP, Coutre SE, et al. Idelalisib and rituximab in relapsed chronic lymphocytic leukemia. *N Engl J Med*. Mar 13 2014;370(11):997-1007. doi:10.1056/NEJMoa1315226

83. Gaillard S, Oaknin A, Ray-Coquard I, et al. Lurbinectedin versus pegylated liposomal doxorubicin or topotecan in patients with platinum-resistant ovarian cancer: A multicenter, randomized, controlled, open-label phase 3 study (CORAIL). *Gynecol Oncol*. Nov 2021;163(2):237-245. doi:10.1016/j.ygyno.2021.08.032

84. Gandhi L, Rodríguez-Abreu D, Gadgeel S, et al. Pembrolizumab plus Chemotherapy in Metastatic Non-Small-Cell Lung Cancer. *N Engl J Med*. May 31 2018;378(22):2078-2092. doi:10.1056/NEJMoa1801005

85. Gianni L, Romieu GH, Lichinitser M, et al. AVEREL: a randomized phase III Trial evaluating bevacizumab in combination with docetaxel and trastuzumab as first-line therapy for HER2-positive locally recurrent/metastatic breast cancer. *J Clin Oncol*. May 10 2013;31(14):1719-25. doi:10.1200/jco.2012.44.7912

86. Gligorov J, Doval D, Bines J, et al. Maintenance capecitabine and bevacizumab versus bevacizumab alone after initial first-line bevacizumab and docetaxel for patients with HER2-negative metastatic breast cancer (IMELDA): a randomised, open-label, phase 3 trial. *Lancet Oncol*. Nov 2014;15(12):1351-60. doi:10.1016/s1470-2045(14)70444-9

87. González-Martín A, Pothuri B, Vergote I, et al. Niraparib in Patients with Newly Diagnosed Advanced Ovarian Cancer. *N Engl J Med*. Dec 19 2019;381(25):2391-2402. doi:10.1056/NEJMoa1910962

88. Gordon AN, Teneriello M, Janicek MF, et al. Phase III trial of induction gemcitabine or paclitaxel plus carboplatin followed by paclitaxel consolidation in ovarian cancer. *Gynecol Oncol*. Dec 2011;123(3):479-85. doi:10.1016/j.ygyno.2011.08.018

89. Gounder MM, Mahoney MR, Van Tine BA, et al. Sorafenib for Advanced and Refractory Desmoid Tumors. *N Engl J Med*. Dec 20 2018;379(25):2417-2428. doi:10.1056/NEJMoa1805052

90. Grosicki S, Simonova M, Spicka I, et al. Once-per-week selinexor, bortezomib, and dexamethasone versus twice-per-week bortezomib and dexamethasone in patients with multiple myeloma (BOSTON): a randomised, open-label, phase 3 trial. *Lancet*. Nov 14 2020;396(10262):1563-1573. doi:10.1016/s0140-6736(20)32292-3

91. Gutzmer R, Stroyakovskiy D, Gogas H, et al. Atezolizumab, vemurafenib, and cobimetinib as first-line treatment for unresectable advanced BRAF(V600) mutation-positive melanoma (IMspire150): primary analysis of the randomised, double-blind, placebo-controlled, phase 3 trial. *Lancet*. Jun 13 2020;395(10240):1835-1844. doi:10.1016/s0140-6736(20)30934-x

92. Hallek M, Fischer K, Fingerle-Rowson G, et al. Addition of rituximab to fludarabine and cyclophosphamide in patients with chronic lymphocytic leukaemia: a randomised, open-label, phase 3 trial. *Lancet*. Oct 2 2010;376(9747):1164-74. doi:10.1016/s0140-6736(10)61381-5

93. Hanna NH, Kaiser R, Sullivan RN, et al. Nintedanib plus pemetrexed versus placebo plus pemetrexed in patients with relapsed or refractory, advanced non-small cell lung cancer (LUME-Lung 2): A randomized, double-blind, phase III trial. *Lung Cancer*. Dec 2016;102:65-73. doi:10.1016/j.lungcan.2016.10.011

94. Harbeck N, Huang CS, Hurvitz S, et al. Afatinib plus vinorelbine versus trastuzumab plus vinorelbine in patients with HER2-overexpressing metastatic breast cancer who had progressed on one previous trastuzumab treatment (LUX-Breast 1): an open-label, randomised, phase 3 trial. *Lancet Oncol*. Mar 2016;17(3):357-366. doi:10.1016/s1470-2045(15)00540-9

95. Hauschild A, Agarwala SS, Trefzer U, et al. Results of a phase III, randomized, placebo-controlled study of sorafenib in combination with carboplatin and paclitaxel as second-line treatment in patients with unresectable stage III or stage IV melanoma. *J Clin Oncol*. Jun 10 2009;27(17):2823-30. doi:10.1200/jco.2007.15.7636

96. Hauschild A, Grob JJ, Demidov LV, et al. Dabrafenib in BRAF-mutated metastatic melanoma: a multicentre, open-label, phase 3 randomised controlled trial. *Lancet*. Jul 28 2012;380(9839):358-65. doi:10.1016/s0140-6736(12)60868-x

97. Hecht JR, Mitchell E, Chidiac T, et al. A randomized phase IIIB trial of chemotherapy, bevacizumab, and panitumumab compared with chemotherapy and bevacizumab alone for metastatic colorectal cancer. *J Clin Oncol*. Feb 10 2009;27(5):672-80. doi:10.1200/jco.2008.19.8135

98. Hensley ML, Miller A, O'Malley DM, et al. Randomized phase III trial of gemcitabine plus docetaxel plus bevacizumab or placebo as first-line treatment for metastatic uterine leiomyosarcoma: an NRG Oncology/Gynecologic Oncology Group study. *J Clin Oncol*. Apr 1 2015;33(10):1180-5. doi:10.1200/jco.2014.58.3781

99. Herbst RS, Sun Y, Eberhardt WE, et al. Vandetanib plus docetaxel versus docetaxel as second-line treatment for patients with advanced non-small-cell lung cancer (ZODIAC): a double-blind, randomised, phase 3 trial. *Lancet Oncol*. Jul 2010;11(7):619-26. doi:10.1016/s1470-2045(10)70132-7

100. Hersh EM, Del Vecchio M, Brown MP, et al. A randomized, controlled phase III trial of nab-Paclitaxel versus dacarbazine in chemotherapy-naïve patients with metastatic melanoma. *Ann Oncol*. Nov 2015;26(11):2267-74. doi:10.1093/annonc/mdv324

101. Horn L, Mansfield AS, Szczęsna A, et al. First-Line Atezolizumab plus Chemotherapy in Extensive-Stage Small-Cell Lung Cancer. *N Engl J Med*. Dec 6 2018;379(23):2220-2229. doi:10.1056/NEJMoa1809064

102. Huang X, Qiu L, Jin J, et al. Ibrutinib versus rituximab in relapsed or refractory chronic lymphocytic leukemia or small lymphocytic lymphoma: a randomized, open-label phase 3 study. *Cancer Med*. Apr 2018;7(4):1043-1055. doi:10.1002/cam4.1337

103. Hutson TE, Escudier B, Esteban E, et al. Randomized phase III trial of temsirolimus versus sorafenib as second-line therapy after sunitinib in patients with metastatic renal cell carcinoma. *J Clin Oncol*. Mar 10 2014;32(8):760-7. doi:10.1200/jco.2013.50.3961

104. Jänne PA, van den Heuvel MM, Barlesi F, et al. Selumetinib Plus Docetaxel Compared With Docetaxel Alone and Progression-Free Survival in Patients With KRAS-Mutant Advanced Non-Small Cell Lung Cancer: The SELECT-1 Randomized Clinical Trial. *Jama*. May 9 2017;317(18):1844-1853. doi:10.1001/jama.2017.3438

105. Jian H, Li W, Ma Z, et al. Intercalating and maintenance gefitinib plus chemotherapy versus chemotherapy alone in selected advanced non-small cell lung cancer with unknown EGFR status. *Sci Rep*. Aug 16 2017;7(1):8483. doi:10.1038/s41598-017-08399-8

106. Johnson BE, Kabbinavar F, Fehrenbacher L, et al. ATLAS: randomized, double-blind, placebo-controlled, phase IIIB trial comparing bevacizumab therapy with or without erlotinib, after completion of chemotherapy, with bevacizumab for first-line treatment of advanced non-small-cell lung cancer. *J Clin Oncol*. Nov 1 2013;31(31):3926-34. doi:10.1200/jco.2012.47.3983

107. Jones RL, Ravi V, Brohl AS, et al. Efficacy and Safety of TRC105 Plus Pazopanib vs Pazopanib Alone for Treatment of Patients With Advanced Angiosarcoma: A Randomized Clinical Trial. *JAMA Oncol*. May 1 2022;8(5):740-747. doi:10.1001/jamaoncol.2021.3547

108. Kang YK, George S, Jones RL, et al. Avapritinib Versus Regorafenib in Locally Advanced Unresectable or Metastatic GI Stromal Tumor: A Randomized, Open-Label Phase III Study. *J Clin Oncol*. Oct 1 2021;39(28):3128-3139. doi:10.1200/jco.21.00217

109. Kang YK, Ryu MH, Yoo C, et al. Resumption of imatinib to control metastatic or unresectable gastrointestinal stromal tumours after failure of imatinib and sunitinib (RIGHT): a randomised, placebo-controlled, phase 3 trial. *Lancet Oncol*. Nov 2013;14(12):1175-82. doi:10.1016/s1470-2045(13)70453-4

110. Kater AP, Owen C, Moreno C, et al. Fixed-Duration Ibrutinib-Venetoclax in Patients with Chronic Lymphocytic Leukemia and Comorbidities. *NEJM Evid*. Jul 2022;1(7):EVIDoa2200006. doi:10.1056/EVIDoa2200006

111. Kaufman B, Mackey JR, Clemens MR, et al. Trastuzumab plus anastrozole versus anastrozole alone for the treatment of postmenopausal women with human epidermal growth factor receptor 2-positive, hormone receptor-positive metastatic breast cancer: results from the randomized phase III TAnDEM study. *J Clin Oncol*. Nov 20 2009;27(33):5529-37. doi:10.1200/jco.2008.20.6847

112. Kim HR, Sugawara S, Lee JS, et al. First-line nivolumab, paclitaxel, carboplatin, and bevacizumab for advanced non-squamous non-small cell lung cancer: Updated survival analysis of the ONO-4538-52/TASUKI-52 randomized controlled trial. *Cancer Med*. Aug 2023;12(16):17061-17067. doi:10.1002/cam4.6348

113. Kristeleit R, Lisyanskaya A, Fedenko A, et al. Rucaparib versus standard-of-care chemotherapy in patients with relapsed ovarian cancer and a deleterious BRCA1 or BRCA2 mutation (ARIEL4): an international, open-label, randomised, phase 3 trial. *Lancet Oncol*. Apr 2022;23(4):465-478. doi:10.1016/s1470-2045(22)00122-x

114. Krop IE, Kim SB, González-Martín A, et al. Trastuzumab emtansine versus treatment of physician's choice for pretreated HER2-positive advanced breast cancer (TH3RESA): a randomised, open-label, phase 3 trial. *Lancet Oncol*. Jun 2014;15(7):689-99. doi:10.1016/s1470-2045(14)70178-0

115. Kropff M, Vogel M, Bisping G, et al. Bortezomib and low-dose dexamethasone with or without continuous low-dose oral cyclophosphamide for primary refractory or relapsed multiple myeloma: a randomized phase III study. *Ann Hematol*. Nov 2017;96(11):1857-1866. doi:10.1007/s00277-017-3065-z

116. Kudo M, Imanaka K, Chida N, et al. Phase III study of sorafenib after transarterial chemoembolisation in Japanese and Korean patients with unresectable hepatocellular carcinoma. *Eur J Cancer*. Sep 2011;47(14):2117-27. doi:10.1016/j.ejca.2011.05.007

117. Kumar SK, Harrison SJ, Cavo M, et al. Venetoclax or placebo in combination with bortezomib and dexamethasone in patients with relapsed or refractory multiple myeloma (BELLINI): a randomised, double-blind, multicentre, phase 3 trial. *Lancet Oncol*. Dec 2020;21(12):1630-1642. doi:10.1016/s1470-2045(20)30525-8

118. Larkin J, Ascierto PA, Dréno B, et al. Combined vemurafenib and cobimetinib in BRAF-mutated melanoma. *N Engl J Med*. Nov 13 2014;371(20):1867-76. doi:10.1056/NEJMoa1408868

119. Lee NY, Ferris RL, Psyrri A, et al. Avelumab plus standard-of-care chemoradiotherapy versus chemoradiotherapy alone in patients with locally advanced squamous cell carcinoma of the head and neck: a randomised, double-blind, placebo-controlled, multicentre, phase 3 trial. *Lancet Oncol*. Apr 2021;22(4):450-462. doi:10.1016/s1470-2045(20)30737-3

120. Lim M, Weller M, Idbaih A, et al. Phase III trial of chemoradiotherapy with temozolomide plus nivolumab or placebo for newly diagnosed glioblastoma with methylated MGMT promoter. *Neuro Oncol*. Nov 2 2022;24(11):1935-1949. doi:10.1093/neuonc/noac116

121. Long GV, Dummer R, Hamid O, et al. Epacadostat plus pembrolizumab versus placebo plus pembrolizumab in patients with unresectable or metastatic melanoma (ECHO-301/KEYNOTE-252): a phase 3, randomised, double-blind study. *Lancet Oncol*. Aug 2019;20(8):1083-1097. doi:10.1016/s1470-2045(19)30274-8

122. Long GV, Stroyakovskiy D, Gogas H, et al. Combined BRAF and MEK inhibition versus BRAF inhibition alone in melanoma. *N Engl J Med*. Nov 13 2014;371(20):1877-88. doi:10.1056/NEJMoa1406037

123. Lonial S, Jacobus S, Fonseca R, et al. Randomized Trial of Lenalidomide Versus Observation in Smoldering Multiple Myeloma. *J Clin Oncol*. Apr 10 2020;38(11):1126-1137. doi:10.1200/jco.19.01740

124. Lu S, Dong X, Jian H, et al. AENEAS: A Randomized Phase III Trial of Aumolertinib Versus Gefitinib as First-Line Therapy for Locally Advanced or MetastaticNon-Small-Cell Lung Cancer With EGFR Exon 19 Deletion or L858R Mutations. *J Clin Oncol*. Sep 20 2022;40(27):3162-3171. doi:10.1200/jco.21.02641

125. Lynch TJ, Patel T, Dreisbach L, et al. Cetuximab and first-line taxane/carboplatin chemotherapy in advanced non-small-cell lung cancer: results of the randomized multicenter phase III trial BMS099. *J Clin Oncol*. Feb 20 2010;28(6):911-7. doi:10.1200/jco.2009.21.9618

126. Mackey JR, Ramos-Vazquez M, Lipatov O, et al. Primary results of ROSE/TRIO-12, a randomized placebo-controlled phase III trial evaluating the addition of ramucirumab to first-line docetaxel chemotherapy in metastatic breast cancer. *J Clin Oncol*. Jan 10 2015;33(2):141-8. doi:10.1200/jco.2014.57.1513

127. Mai HQ, Chen QY, Chen D, et al. Toripalimab or placebo plus chemotherapy as first-line treatment in advanced nasopharyngeal carcinoma: a multicenter randomized phase 3 trial. *Nat Med*. Sep 2021;27(9):1536-1543. doi:10.1038/s41591-021-01444-0

128. Makker V, Colombo N, Casado Herráez A, et al. Lenvatinib plus Pembrolizumab for Advanced Endometrial Cancer. *N Engl J Med*. Feb 3 2022;386(5):437-448. doi:10.1056/NEJMoa2108330

129. Maloney DG, Ogura M, Fukuhara N, et al. A phase 3 randomized study (HOMER) of ofatumumab vs rituximab in iNHL relapsed after rituximab-containing therapy. *Blood Adv*. Aug 25 2020;4(16):3886-3893. doi:10.1182/bloodadvances.2020001942

130. Matasar MJ, Capra M, Özcan M, et al. Copanlisib plus rituximab versus placebo plus rituximab in patients with relapsed indolent non-Hodgkin lymphoma (CHRONOS-3): a double-blind, randomised, placebo-controlled, phase 3 trial. *Lancet Oncol*. May 2021;22(5):678-689. doi:10.1016/s1470-2045(21)00145-5

131. Mateos MV, Blacklock H, Schjesvold F, et al. Pembrolizumab plus pomalidomide and dexamethasone for patients with relapsed or refractory multiple myeloma (KEYNOTE-183): a randomised, open-label, phase 3 trial. *Lancet Haematol*. Sep 2019;6(9):e459-e469. doi:10.1016/s2352-3026(19)30110-3

132. Mateos MV, Dimopoulos MA, Cavo M, et al. Daratumumab plus Bortezomib, Melphalan, and Prednisone for Untreated Myeloma. *N Engl J Med*. Feb 8 2018;378(6):518-528. doi:10.1056/NEJMoa1714678

133. McCarthy PL, Owzar K, Hofmeister CC, et al. Lenalidomide after stem-cell transplantation for multiple myeloma. *N Engl J Med*. May 10 2012;366(19):1770-81. doi:10.1056/NEJMoa1114083

134. McCleary NJ, Hubbard J, Mahoney MR, et al. Challenges of conducting a prospective clinical trial for older patients: Lessons learned from NCCTG N0949 (alliance). *J Geriatr Oncol*. Jan 2018;9(1):24-31. doi:10.1016/j.jgo.2017.08.005

135. Mehta RS, Barlow WE, Albain KS, et al. Combination anastrozole and fulvestrant in metastatic breast cancer. *N Engl J Med*. Aug 2 2012;367(5):435-44. doi:10.1056/NEJMoa1201622

136. Mellinghoff IK, van den Bent MJ, Blumenthal DT, et al. Vorasidenib in IDH1- or IDH2-Mutant Low-Grade Glioma. *N Engl J Med*. Aug 17 2023;389(7):589-601. doi:10.1056/NEJMoa2304194

137. Merseburger AS, Attard G, Åström L, et al. Continuous enzalutamide after progression of metastatic castration-resistant prostate cancer treated with docetaxel (PRESIDE): an international, randomised, phase 3b study. *Lancet Oncol*. Nov 2022;23(11):1398-1408. doi:10.1016/s1470-2045(22)00560-5

138. Miguel JS, Weisel K, Moreau P, et al. Pomalidomide plus low-dose dexamethasone versus high-dose dexamethasone alone for patients with relapsed and refractory multiple myeloma (MM-003): a randomised, open-label, phase 3 trial. *Lancet Oncol*. Oct 2013;14(11):1055-1066. doi:10.1016/s1470-2045(13)70380-2

139. Miles D, Gligorov J, André F, et al. Primary results from IMpassion131, a double-blind, placebo-controlled, randomised phase III trial of first-line paclitaxel with or without atezolizumab for unresectable locally advanced/metastatic triple-negative breast cancer. *Ann Oncol*. Aug 2021;32(8):994-1004. doi:10.1016/j.annonc.2021.05.801

140. Modi S, Jacot W, Yamashita T, et al. Trastuzumab Deruxtecan in Previously Treated HER2-Low Advanced Breast Cancer. *N Engl J Med*. Jul 7 2022;387(1):9-20. doi:10.1056/NEJMoa2203690

141. Mok T, Nakagawa K, Park K, et al. Nivolumab Plus Chemotherapy in Epidermal Growth Factor Receptor-Mutated Metastatic Non-Small-Cell Lung Cancer After Disease Progression on Epidermal Growth Factor Receptor Tyrosine Kinase Inhibitors: Final Results of CheckMate 722. *J Clin Oncol*. Apr 10 2024;42(11):1252-1264. doi:10.1200/jco.23.01017

142. Monk BJ, Herzog TJ, Kaye SB, et al. Trabectedin plus pegylated liposomal Doxorubicin in recurrent ovarian cancer. *J Clin Oncol*. Jul 1 2010;28(19):3107-14. doi:10.1200/jco.2009.25.4037

143. Monk BJ, Toita T, Wu X, et al. Durvalumab versus placebo with chemoradiotherapy for locally advanced cervical cancer (CALLA): a randomised, double-blind, phase 3 trial. *Lancet Oncol*. Dec 2023;24(12):1334-1348. doi:10.1016/s1470-2045(23)00479-5

144. Moore KN, Bookman M, Sehouli J, et al. Atezolizumab, Bevacizumab, and Chemotherapy for Newly Diagnosed Stage III or IV Ovarian Cancer: Placebo-Controlled Randomized Phase III Trial (IMagyn050/GOG 3015/ENGOT-OV39). *J Clin Oncol*. Jun 10 2021;39(17):1842-1855. doi:10.1200/jco.21.00306

145. Moore KN, Oza AM, Colombo N, et al. Phase III, randomized trial of mirvetuximab soravtansine versus chemotherapy in patients with platinum-resistant ovarian cancer: primary analysis of FORWARD I. *Ann Oncol*. Jun 2021;32(6):757-765. doi:10.1016/j.annonc.2021.02.017

146. Moreau P, Dimopoulos MA, Mikhael J, et al. Isatuximab, carfilzomib, and dexamethasone in relapsed multiple myeloma (IKEMA): a multicentre, open-label, randomised phase 3 trial. *Lancet*. Jun 19 2021;397(10292):2361-2371. doi:10.1016/s0140-6736(21)00592-4

147. Moreau P, Masszi T, Grzasko N, et al. Oral Ixazomib, Lenalidomide, and Dexamethasone for Multiple Myeloma. *N Engl J Med*. Apr 28 2016;374(17):1621-34. doi:10.1056/NEJMoa1516282

148. Motzer RJ, Escudier B, Oudard S, et al. Efficacy of everolimus in advanced renal cell carcinoma: a double-blind, randomised, placebo-controlled phase III trial. *Lancet*. Aug 9 2008;372(9637):449-56. doi:10.1016/s0140-6736(08)61039-9

149. Motzer RJ, Porta C, Vogelzang NJ, et al. Dovitinib versus sorafenib for third-line targeted treatment of patients with metastatic renal cell carcinoma: an open-label, randomised phase 3 trial. *Lancet Oncol*. Mar 2014;15(3):286-96. doi:10.1016/s1470-2045(14)70030-0

150. Motzer RJ, Tannir NM, McDermott DF, et al. Nivolumab plus Ipilimumab versus Sunitinib in Advanced Renal-Cell Carcinoma. *N Engl J Med*. Apr 5 2018;378(14):1277-1290. doi:10.1056/NEJMoa1712126

151. Nastoupil LJ, Hess G, Pavlovsky MA, et al. Phase 3 SELENE study: ibrutinib plus BR/R-CHOP in previously treated patients with follicular or marginal zone lymphoma. *Blood Adv*. Nov 28 2023;7(22):7141-7150. doi:10.1182/bloodadvances.2023010298

152. Natale RB, Thongprasert S, Greco FA, et al. Phase III trial of vandetanib compared with erlotinib in patients with previously treated advanced non-small-cell lung cancer. *J Clin Oncol*. Mar 10 2011;29(8):1059-66. doi:10.1200/jco.2010.28.5981

153. Nowakowski GS, Chiappella A, Gascoyne RD, et al. ROBUST: A Phase III Study of Lenalidomide Plus R-CHOP Versus Placebo Plus R-CHOP in Previously Untreated Patients With ABC-Type Diffuse Large B-Cell Lymphoma. *J Clin Oncol*. Apr 20 2021;39(12):1317-1328. doi:10.1200/jco.20.01366

154. Orlowski RZ, Nagler A, Sonneveld P, et al. Randomized phase III study of pegylated liposomal doxorubicin plus bortezomib compared with bortezomib alone in relapsed or refractory multiple myeloma: combination therapy improves time to progression. *J Clin Oncol*. Sep 1 2007;25(25):3892-901. doi:10.1200/jco.2006.10.5460

155. Österborg A, Udvardy M, Zaritskey A, et al. Phase III, randomized study of ofatumumab versus physicians' choice of therapy and standard versus extended-length ofatumumab in patients with bulky fludarabine-refractory chronic lymphocytic leukemia. *Leuk Lymphoma*. Sep 2016;57(9):2037-46. doi:10.3109/10428194.2015.1122783

156. Pal SK, Albiges L, Tomczak P, et al. Atezolizumab plus cabozantinib versus cabozantinib monotherapy for patients with renal cell carcinoma after progression with previous immune checkpoint inhibitor treatment (CONTACT-03): a multicentre, randomised, open-label, phase 3 trial. *Lancet*. Jul 15 2023;402(10397):185-195. doi:10.1016/s0140-6736(23)00922-4

157. Pavel ME, Hainsworth JD, Baudin E, et al. Everolimus plus octreotide long-acting repeatable for the treatment of advanced neuroendocrine tumours associated with carcinoid syndrome (RADIANT-2): a randomised, placebo-controlled, phase 3 study. *Lancet*. Dec 10 2011;378(9808):2005-2012. doi:10.1016/s0140-6736(11)61742-x

158. Paz-Ares L, de Marinis F, Dediu M, et al. Maintenance therapy with pemetrexed plus best supportive care versus placebo plus best supportive care after induction therapy with pemetrexed plus cisplatin for advanced non-squamous non-small-cell lung cancer (PARAMOUNT): a double-blind, phase 3, randomised controlled trial. *Lancet Oncol*. Mar 2012;13(3):247-55. doi:10.1016/s1470-2045(12)70063-3

159. Paz-Ares L, Luft A, Vicente D, et al. Pembrolizumab plus Chemotherapy for Squamous Non-Small-Cell Lung Cancer. *N Engl J Med*. Nov 22 2018;379(21):2040-2051. doi:10.1056/NEJMoa1810865

160. Peeters M, Price TJ, Cervantes A, et al. Randomized phase III study of panitumumab with fluorouracil, leucovorin, and irinotecan (FOLFIRI) compared with FOLFIRI alone as second-line treatment in patients with metastatic colorectal cancer. *J Clin Oncol*. Nov 1 2010;28(31):4706-13. doi:10.1200/jco.2009.27.6055

161. Pour L, Szarejko M, Bila J, et al. Efficacy and safety of melflufen plus daratumumab and dexamethasone in relapsed/refractory multiple myeloma: results from the randomized, open-label, phase III LIGHTHOUSE study. *Haematologica*. Mar 1 2024;109(3):895-905. doi:10.3324/haematol.2023.283509

162. Press OW, Unger JM, Rimsza LM, et al. Phase III randomized intergroup trial of CHOP plus rituximab compared with CHOP chemotherapy plus (131)iodine-tositumomab for previously untreated follicular non-Hodgkin lymphoma: SWOG S0016. *J Clin Oncol*. Jan 20 2013;31(3):314-20. doi:10.1200/jco.2012.42.4101

163. Pujade-Lauraine E, Hilpert F, Weber B, et al. Bevacizumab combined with chemotherapy for platinum-resistant recurrent ovarian cancer: The AURELIA open-label randomized phase III trial. *J Clin Oncol*. May 1 2014;32(13):1302-8. doi:10.1200/jco.2013.51.4489

164. Qin S, Chan SL, Gu S, et al. Camrelizumab plus rivoceranib versus sorafenib as first-line therapy for unresectable hepatocellular carcinoma (CARES-310): a randomised, open-label, international phase 3 study. *Lancet*. Sep 30 2023;402(10408):1133-1146. doi:10.1016/s0140-6736(23)00961-3

165. Qin S, Li J, Wang L, et al. Efficacy and Tolerability of First-Line Cetuximab Plus Leucovorin, Fluorouracil, and Oxaliplatin (FOLFOX-4) Versus FOLFOX-4 in Patients With RAS Wild-Type Metastatic Colorectal Cancer: The Open-Label, Randomized, Phase III TAILOR Trial. *J Clin Oncol*. Oct 20 2018;36(30):3031-3039. doi:10.1200/jco.2018.78.3183

166. Ramalingam SS, Jänne PA, Mok T, et al. Dacomitinib versus erlotinib in patients with advanced-stage, previously treated non-small-cell lung cancer (ARCHER 1009): a randomised, double-blind, phase 3 trial. *Lancet Oncol*. Nov 2014;15(12):1369-78. doi:10.1016/s1470-2045(14)70452-8

167. Raymond E, Dahan L, Raoul JL, et al. Sunitinib malate for the treatment of pancreatic neuroendocrine tumors. *N Engl J Med*. Feb 10 2011;364(6):501-13. doi:10.1056/NEJMoa1003825

168. Reck M, Kaiser R, Mellemgaard A, et al. Docetaxel plus nintedanib versus docetaxel plus placebo in patients with previously treated non-small-cell lung cancer (LUME-Lung 1): a phase 3, double-blind, randomised controlled trial. *Lancet Oncol*. Feb 2014;15(2):143-55. doi:10.1016/s1470-2045(13)70586-2

169. Reck M, Rodríguez-Abreu D, Robinson AG, et al. Pembrolizumab versus Chemotherapy for PD-L1-Positive Non-Small-Cell Lung Cancer. *N Engl J Med*. Nov 10 2016;375(19):1823-1833. doi:10.1056/NEJMoa1606774

170. Ren S, Feng J, Ma S, et al. KEYNOTE-033: Randomized phase 3 study of pembrolizumab vs docetaxel in previously treated, PD-L1-positive, advanced NSCLC. *Int J Cancer*. Aug 1 2023;153(3):623-634. doi:10.1002/ijc.34532

171. Richardson PG, Oriol A, Beksac M, et al. Pomalidomide, bortezomib, and dexamethasone for patients with relapsed or refractory multiple myeloma previously treated with lenalidomide (OPTIMISMM): a randomised, open-label, phase 3 trial. *Lancet Oncol*. Jun 2019;20(6):781-794. doi:10.1016/s1470-2045(19)30152-4

172. Rini BI, Bellmunt J, Clancy J, et al. Randomized phase III trial of temsirolimus and bevacizumab versus interferon alfa and bevacizumab in metastatic renal cell carcinoma: INTORACT trial. *J Clin Oncol*. Mar 10 2014;32(8):752-9. doi:10.1200/jco.2013.50.5305

173. Rini BI, Pal SK, Escudier BJ, et al. Tivozanib versus sorafenib in patients with advanced renal cell carcinoma (TIVO-3): a phase 3, multicentre, randomised, controlled, open-label study. *Lancet Oncol*. Jan 2020;21(1):95-104. doi:10.1016/s1470-2045(19)30735-1

174. Rini BI, Plimack ER, Stus V, et al. Pembrolizumab plus Axitinib versus Sunitinib for Advanced Renal-Cell Carcinoma. *N Engl J Med*. Mar 21 2019;380(12):1116-1127. doi:10.1056/NEJMoa1816714

175. Rini BI, Powles T, Atkins MB, et al. Atezolizumab plus bevacizumab versus sunitinib in patients with previously untreated metastatic renal cell carcinoma (IMmotion151): a multicentre, open-label, phase 3, randomised controlled trial. *Lancet*. Jun 15 2019;393(10189):2404-2415. doi:10.1016/s0140-6736(19)30723-8

176. Robak T, Huang H, Jin J, et al. Bortezomib-based therapy for newly diagnosed mantle-cell lymphoma. *N Engl J Med*. Mar 5 2015;372(10):944-53. doi:10.1056/NEJMoa1412096

177. Robak T, Warzocha K, Govind Babu K, et al. Ofatumumab plus fludarabine and cyclophosphamide in relapsed chronic lymphocytic leukemia: results from the COMPLEMENT 2 trial. *Leuk Lymphoma*. May 2017;58(5):1084-1093. doi:10.1080/10428194.2016.1233536

178. Robert NJ, Saleh MN, Paul D, et al. Sunitinib plus paclitaxel versus bevacizumab plus paclitaxel for first-line treatment of patients with advanced breast cancer: a phase III, randomized, open-label trial. *Clin Breast Cancer*. Apr 2011;11(2):82-92. doi:10.1016/j.clbc.2011.03.005

179. Rudin CM, Awad MM, Navarro A, et al. Pembrolizumab or Placebo Plus Etoposide and Platinum as First-Line Therapy for Extensive-Stage Small-Cell Lung Cancer: Randomized, Double-Blind, Phase III KEYNOTE-604 Study. *J Clin Oncol*. Jul 20 2020;38(21):2369-2379. doi:10.1200/jco.20.00793

180. Rugo HS, Im SA, Cardoso F, et al. Efficacy of Margetuximab vs Trastuzumab in Patients With Pretreated ERBB2-Positive Advanced Breast Cancer: A Phase 3 Randomized Clinical Trial. *JAMA Oncol*. Apr 1 2021;7(4):573-584. doi:10.1001/jamaoncol.2020.7932

181. Rummel MJ, Janssens A, MacDonald D, et al. A phase 3, randomized study of ofatumumab combined with bendamustine in rituximab-refractory iNHL (COMPLEMENT A + B study). *Br J Haematol*. Jun 2021;193(6):1123-1133. doi:10.1111/bjh.17420

182. Saad F, Fizazi K, Jinga V, et al. Orteronel plus prednisone in patients with chemotherapy-naive metastatic castration-resistant prostate cancer (ELM-PC 4): a double-blind, multicentre, phase 3, randomised, placebo-controlled trial. *Lancet Oncol*. Mar 2015;16(3):338-48. doi:10.1016/s1470-2045(15)70027-6

183. Saltz L, Badarinath S, Dakhil S, et al. Phase III trial of cetuximab, bevacizumab, and 5-fluorouracil/leucovorin vs. FOLFOX-bevacizumab in colorectal cancer. *Clin Colorectal Cancer*. Jun 2012;11(2):101-11. doi:10.1016/j.clcc.2011.05.006

184. San-Miguel JF, Hungria VT, Yoon SS, et al. Panobinostat plus bortezomib and dexamethasone versus placebo plus bortezomib and dexamethasone in patients with relapsed or relapsed and refractory multiple myeloma: a multicentre, randomised, double-blind phase 3 trial. *Lancet Oncol*. Oct 2014;15(11):1195-206. doi:10.1016/s1470-2045(14)70440-1

185. Saura C, Oliveira M, Feng YH, et al. Neratinib Plus Capecitabine Versus Lapatinib Plus Capecitabine in HER2-Positive Metastatic Breast Cancer Previously Treated With ≥ 2 HER2-Directed Regimens: Phase III NALA Trial. *J Clin Oncol*. Sep 20 2020;38(27):3138-3149. doi:10.1200/jco.20.00147

186. Scagliotti GV, Kosmidis P, de Marinis F, et al. Zoledronic acid in patients with stage IIIA/B NSCLC: results of a randomized, phase III study. *Ann Oncol*. Aug 2012;23(8):2082-2087. doi:10.1093/annonc/mds128

187. Schjesvold FH, Dimopoulos MA, Delimpasi S, et al. Melflufen or pomalidomide plus dexamethasone for patients with multiple myeloma refractory to lenalidomide (OCEAN): a randomised, head-to-head, open-label, phase 3 study. *Lancet Haematol*. Feb 2022;9(2):e98-e110. doi:10.1016/s2352-3026(21)00381-1

188. Schmid P, Adams S, Rugo HS, et al. Atezolizumab and Nab-Paclitaxel in Advanced Triple-Negative Breast Cancer. *N Engl J Med*. Nov 29 2018;379(22):2108-2121. doi:10.1056/NEJMoa1809615

189. Schmoll HJ, Cunningham D, Sobrero A, et al. Cediranib with mFOLFOX6 versus bevacizumab with mFOLFOX6 as first-line treatment for patients with advanced colorectal cancer: a double-blind, randomized phase III study (HORIZON III). *J Clin Oncol*. Oct 10 2012;30(29):3588-95. doi:10.1200/jco.2012.42.5355

190. Schuler M, Yang JC, Park K, et al. Afatinib beyond progression in patients with non-small-cell lung cancer following chemotherapy, erlotinib/gefitinib and afatinib: phase III randomized LUX-Lung 5 trial. *Ann Oncol*. Mar 2016;27(3):417-23. doi:10.1093/annonc/mdv597

191. Schwartzberg LS, Franco SX, Florance A, O'Rourke L, Maltzman J, Johnston S. Lapatinib plus letrozole as first-line therapy for HER-2+ hormone receptor-positive metastatic breast cancer. *Oncologist*. 2010;15(2):122-9. doi:10.1634/theoncologist.2009-0240

192. Sehn LH, Chua N, Mayer J, et al. Obinutuzumab plus bendamustine versus bendamustine monotherapy in patients with rituximab-refractory indolent non-Hodgkin lymphoma (GADOLIN): a randomised, controlled, open-label, multicentre, phase 3 trial. *Lancet Oncol*. Aug 2016;17(8):1081-1093. doi:10.1016/s1470-2045(16)30097-3

193. Seidman AD, Brufsky A, Ansari RH, et al. Phase III trial of gemcitabine plus docetaxel versus capecitabine plus docetaxel with planned crossover to the alternate single agent in metastatic breast cancer. *Ann Oncol*. May 2011;22(5):1094-1101. doi:10.1093/annonc/mdq578

194. Sequist LV, Yang JC, Yamamoto N, et al. Phase III study of afatinib or cisplatin plus pemetrexed in patients with metastatic lung adenocarcinoma with EGFR mutations. *J Clin Oncol*. Sep 20 2013;31(27):3327-34. doi:10.1200/jco.2012.44.2806

195. Seymour JF, Pfreundschuh M, Trnĕný M, et al. R-CHOP with or without bevacizumab in patients with previously untreated diffuse large B-cell lymphoma: final MAIN study outcomes. *Haematologica*. Aug 2014;99(8):1343-9. doi:10.3324/haematol.2013.100818

196. Shanafelt TD, Wang XV, Kay NE, et al. Ibrutinib-Rituximab or Chemoimmunotherapy for Chronic Lymphocytic Leukemia. *N Engl J Med*. Aug 1 2019;381(5):432-443. doi:10.1056/NEJMoa1817073

197. Shaw AT, Bauer TM, de Marinis F, et al. First-Line Lorlatinib or Crizotinib in Advanced ALK-Positive Lung Cancer. *N Engl J Med*. Nov 19 2020;383(21):2018-2029. doi:10.1056/NEJMoa2027187

198. Shitara K, Özgüroğlu M, Bang YJ, et al. Pembrolizumab versus paclitaxel for previously treated, advanced gastric or gastro-oesophageal junction cancer (KEYNOTE-061): a randomised, open-label, controlled, phase 3 trial. *Lancet*. Jul 14 2018;392(10142):123-133. doi:10.1016/s0140-6736(18)31257-1

199. Small EJ, Schellhammer PF, Higano CS, et al. Placebo-controlled phase III trial of immunologic therapy with sipuleucel-T (APC8015) in patients with metastatic, asymptomatic hormone refractory prostate cancer. *J Clin Oncol*. Jul 1 2006;24(19):3089-94. doi:10.1200/jco.2005.04.5252

200. Smit EF, Wu YL, Gervais R, et al. A randomized, double-blind, phase III study comparing two doses of erlotinib for second-line treatment of current smokers with advanced non-small-cell lung cancer (CurrentS). *Lung Cancer*. Sep 2016;99:94-101. doi:10.1016/j.lungcan.2016.06.019

201. Solomon BJ, Mok T, Kim DW, et al. First-line crizotinib versus chemotherapy in ALK-positive lung cancer. *N Engl J Med*. Dec 4 2014;371(23):2167-77. doi:10.1056/NEJMoa1408440

202. Soria JC, Felip E, Cobo M, et al. Afatinib versus erlotinib as second-line treatment of patients with advanced squamous cell carcinoma of the lung (LUX-Lung 8): an open-label randomised controlled phase 3 trial. *Lancet Oncol*. Aug 2015;16(8):897-907. doi:10.1016/s1470-2045(15)00006-6

203. Stewart AK, Rajkumar SV, Dimopoulos MA, et al. Carfilzomib, lenalidomide, and dexamethasone for relapsed multiple myeloma. *N Engl J Med*. Jan 8 2015;372(2):142-52. doi:10.1056/NEJMoa1411321

204. Strosberg J, El-Haddad G, Wolin E, et al. Phase 3 Trial of (177)Lu-Dotatate for Midgut Neuroendocrine Tumors. *N Engl J Med*. Jan 12 2017;376(2):125-135. doi:10.1056/NEJMoa1607427

205. Sun JM, Shen L, Shah MA, et al. Pembrolizumab plus chemotherapy versus chemotherapy alone for first-line treatment of advanced oesophageal cancer (KEYNOTE-590): a randomised, placebo-controlled, phase 3 study. *Lancet*. Aug 28 2021;398(10302):759-771. doi:10.1016/s0140-6736(21)01234-4

206. Tempero M, Oh DY, Tabernero J, et al. Ibrutinib in combination with nab-paclitaxel and gemcitabine for first-line treatment of patients with metastatic pancreatic adenocarcinoma: phase III RESOLVE study. *Ann Oncol*. May 2021;32(5):600-608. doi:10.1016/j.annonc.2021.01.070

207. Turner NC, Ro J, André F, et al. Palbociclib in Hormone-Receptor-Positive Advanced Breast Cancer. *N Engl J Med*. Jul 16 2015;373(3):209-19. doi:10.1056/NEJMoa1505270

208. Usmani SZ, Schjesvold F, Oriol A, et al. Pembrolizumab plus lenalidomide and dexamethasone for patients with treatment-naive multiple myeloma (KEYNOTE-185): a randomised, open-label, phase 3 trial. *Lancet Haematol*. Sep 2019;6(9):e448-e458. doi:10.1016/s2352-3026(19)30109-7

209. Van Cutsem E, Köhne CH, Hitre E, et al. Cetuximab and chemotherapy as initial treatment for metastatic colorectal cancer. *N Engl J Med*. Apr 2 2009;360(14):1408-17. doi:10.1056/NEJMoa0805019

210. Van Cutsem E, Peeters M, Siena S, et al. Open-label phase III trial of panitumumab plus best supportive care compared with best supportive care alone in patients with chemotherapy-refractory metastatic colorectal cancer. *J Clin Oncol*. May 1 2007;25(13):1658-64. doi:10.1200/jco.2006.08.1620

211. van der Graaf WT, Blay JY, Chawla SP, et al. Pazopanib for metastatic soft-tissue sarcoma (PALETTE): a randomised, double-blind, placebo-controlled phase 3 trial. *Lancet*. May 19 2012;379(9829):1879-86. doi:10.1016/s0140-6736(12)60651-5

212. van Imhoff GW, McMillan A, Matasar MJ, et al. Ofatumumab Versus Rituximab Salvage Chemoimmunotherapy in Relapsed or Refractory Diffuse Large B-Cell Lymphoma: The ORCHARRD Study. *J Clin Oncol*. Feb 10 2017;35(5):544-551. doi:10.1200/jco.2016.69.0198

213. Vitolo U, Ladetto M, Boccomini C, et al. Rituximab maintenance compared with observation after brief first-line R-FND chemoimmunotherapy with rituximab consolidation in patients age older than 60 years with advanced follicular lymphoma: a phase III randomized study by the Fondazione Italiana Linfomi. *J Clin Oncol*. Sep 20 2013;31(27):3351-9. doi:10.1200/jco.2012.44.8290

214. Vitolo U, Trněný M, Belada D, et al. Obinutuzumab or Rituximab Plus Cyclophosphamide, Doxorubicin, Vincristine, and Prednisone in Previously Untreated Diffuse Large B-Cell Lymphoma. *J Clin Oncol*. Nov 1 2017;35(31):3529-3537. doi:10.1200/jco.2017.73.3402

215. von Minckwitz G, Puglisi F, Cortes J, et al. Bevacizumab plus chemotherapy versus chemotherapy alone as second-line treatment for patients with HER2-negative locally recurrent or metastatic breast cancer after first-line treatment with bevacizumab plus chemotherapy (TANIA): an open-label, randomised phase 3 trial. *Lancet Oncol*. Oct 2014;15(11):1269-78. doi:10.1016/s1470-2045(14)70439-5

216. Vose JM, Carter S, Burns LJ, et al. Phase III randomized study of rituximab/carmustine, etoposide, cytarabine, and melphalan (BEAM) compared with iodine-131 tositumomab/BEAM with autologous hematopoietic cell transplantation for relapsed diffuse large B-cell lymphoma: results from the BMT CTN 0401 trial. *J Clin Oncol*. May 1 2013;31(13):1662-8. doi:10.1200/jco.2012.45.9453

217. Wang J, Lu S, Yu X, et al. Tislelizumab Plus Chemotherapy vs Chemotherapy Alone as First-line Treatment for Advanced Squamous Non-Small-Cell Lung Cancer: A Phase 3 Randomized Clinical Trial. *JAMA Oncol*. May 1 2021;7(5):709-717. doi:10.1001/jamaoncol.2021.0366

218. Wang ML, Jurczak W, Jerkeman M, et al. Ibrutinib plus Bendamustine and Rituximab in Untreated Mantle-Cell Lymphoma. *N Engl J Med*. Jun 30 2022;386(26):2482-2494. doi:10.1056/NEJMoa2201817

219. Wang ZX, Cui C, Yao J, et al. Toripalimab plus chemotherapy in treatment-naïve, advanced esophageal squamous cell carcinoma (JUPITER-06): A multi-center phase 3 trial. *Cancer Cell*. Mar 14 2022;40(3):277-288.e3. doi:10.1016/j.ccell.2022.02.007

220. Weber DM, Chen C, Niesvizky R, et al. Lenalidomide plus dexamethasone for relapsed multiple myeloma in North America. *N Engl J Med*. Nov 22 2007;357(21):2133-42. doi:10.1056/NEJMoa070596

221. West H, McCleod M, Hussein M, et al. Atezolizumab in combination with carboplatin plus nab-paclitaxel chemotherapy compared with chemotherapy alone as first-line treatment for metastatic non-squamous non-small-cell lung cancer (IMpower130): a multicentre, randomised, open-label, phase 3 trial. *Lancet Oncol*. Jul 2019;20(7):924-937. doi:10.1016/s1470-2045(19)30167-6

222. Wu X, Liu J, An R, et al. First-line bevacizumab plus chemotherapy in Chinese patients with stage III/IV epithelial ovarian cancer, fallopian tube cancer or primary peritoneal cancer: a phase III randomized controlled trial. *J Gynecol Oncol*. Sep 2024;35(5):e99. doi:10.3802/jgo.2024.35.e99

223. Wu YL, Lee JS, Thongprasert S, et al. Intercalated combination of chemotherapy and erlotinib for patients with advanced stage non-small-cell lung cancer (FASTACT-2): a randomised, double-blind trial. *Lancet Oncol*. Jul 2013;14(8):777-86. doi:10.1016/s1470-2045(13)70254-7

224. Wu YL, Zhou C, Hu CP, et al. Afatinib versus cisplatin plus gemcitabine for first-line treatment of Asian patients with advanced non-small-cell lung cancer harbouring EGFR mutations (LUX-Lung 6): an open-label, randomised phase 3 trial. *Lancet Oncol*. Feb 2014;15(2):213-22. doi:10.1016/s1470-2045(13)70604-1

225. Wu YL, Zhou C, Liam CK, et al. First-line erlotinib versus gemcitabine/cisplatin in patients with advanced EGFR mutation-positive non-small-cell lung cancer: analyses from the phase III, randomized, open-label, ENSURE study. *Ann Oncol*. Sep 2015;26(9):1883-1889. doi:10.1093/annonc/mdv270

226. Xu B, Hu X, Li W, et al. Palbociclib plus letrozole versus placebo plus letrozole in Asian postmenopausal women with oestrogen receptor-positive/human epidermal growth factor receptor 2-negative advanced breast cancer: Primary results from PALOMA-4. *Eur J Cancer*. Nov 2022;175:236-245. doi:10.1016/j.ejca.2022.08.012

227. Xu B, Li W, Zhang Q, et al. Pertuzumab, trastuzumab, and docetaxel for Chinese patients with previously untreated HER2-positive locally recurrent or metastatic breast cancer (PUFFIN): a phase III, randomized, double-blind, placebo-controlled study. *Breast Cancer Res Treat*. Aug 2020;182(3):689-697. doi:10.1007/s10549-020-05728-w

228. Xu RH, Zhang Y, Pan H, et al. Efficacy and safety of weekly paclitaxel with or without ramucirumab as second-line therapy for the treatment of advanced gastric or gastroesophageal junction adenocarcinoma (RAINBOW-Asia): a randomised, multicentre, double-blind, phase 3 trial. *Lancet Gastroenterol Hepatol*. Dec 2021;6(12):1015-1024. doi:10.1016/s2468-1253(21)00313-7

229. Yang JC, Han B, De La Mora Jiménez E, et al. Pembrolizumab With or Without Lenvatinib for First-Line Metastatic NSCLC With Programmed Cell Death-Ligand 1 Tumor Proportion Score of at least 1% (LEAP-007): A Randomized, Double-Blind, Phase 3 Trial. *J Thorac Oncol*. Jun 2024;19(6):941-953. doi:10.1016/j.jtho.2023.12.023

230. Yang Y, Wang Z, Fang J, et al. Efficacy and Safety of Sintilimab Plus Pemetrexed and Platinum as First-Line Treatment for Locally Advanced or Metastatic Nonsquamous NSCLC: a Randomized, Double-Blind, Phase 3 Study (Oncology pRogram by InnovENT anti-PD-1-11). *J Thorac Oncol*. Oct 2020;15(10):1636-1646. doi:10.1016/j.jtho.2020.07.014

231. Yao JC, Guthrie KA, Moran C, et al. Phase III Prospective Randomized Comparison Trial of Depot Octreotide Plus Interferon Alfa-2b Versus Depot Octreotide Plus Bevacizumab in Patients With Advanced Carcinoid Tumors: SWOG S0518. *J Clin Oncol*. May 20 2017;35(15):1695-1703. doi:10.1200/jco.2016.70.4072

232. Yao JC, Shah MH, Ito T, et al. Everolimus for advanced pancreatic neuroendocrine tumors. *N Engl J Med*. Feb 10 2011;364(6):514-23. doi:10.1056/NEJMoa1009290

233. Ychou M, Hohenberger W, Thezenas S, et al. A randomized phase III study comparing adjuvant 5-fluorouracil/folinic acid with FOLFIRI in patients following complete resection of liver metastases from colorectal cancer. *Ann Oncol*. Dec 2009;20(12):1964-70. doi:10.1093/annonc/mdp236

234. Yu AL, Gilman AL, Ozkaynak MF, et al. Anti-GD2 antibody with GM-CSF, interleukin-2, and isotretinoin for neuroblastoma. *N Engl J Med*. Sep 30 2010;363(14):1324-34. doi:10.1056/NEJMoa0911123

235. Zhou C, Tang KJ, Cho BC, et al. Amivantamab plus Chemotherapy in NSCLC with EGFR Exon 20 Insertions. *N Engl J Med*. Nov 30 2023;389(22):2039-2051. doi:10.1056/NEJMoa2306441

236. Zhou C, Wu L, Fan Y, et al. Sintilimab Plus Platinum and Gemcitabine as First-Line Treatment for Advanced or Metastatic Squamous NSCLC: Results From a Randomized, Double-Blind, Phase 3 Trial (ORIENT-12). *J Thorac Oncol*. Sep 2021;16(9):1501-1511. doi:10.1016/j.jtho.2021.04.011

237. Zinner RG, Obasaju CK, Spigel DR, et al. PRONOUNCE: randomized, open-label, phase III study of first-line pemetrexed + carboplatin followed by maintenance pemetrexed versus paclitaxel + carboplatin + bevacizumab followed by maintenance bevacizumab in patients ith advanced nonsquamous non-small-cell lung cancer. *J Thorac Oncol*. Jan 2015;10(1):134-42. doi:10.1097/jto.0000000000000366
